# Adipocyte *Gata3* Is a Diet-Inducible Driver of Pathological Adipose Remodeling and Systemic Metabolic Disease

**DOI:** 10.3390/cells15181644

**Published:** 2026-09-10

**Authors:** Minghui Qin, Fuqiang Li, Mingjie Yang, Lai Wang, Behrooz G. Sharifi, Prediman K. Shah

**Affiliations:** 1 The Oppenheimer Atherosclerosis Research Center, Cedars Sinai Heart Institute Cedars-Sinai Med. Ctr., Los Angeles, CA 90048, USA; minghui.qin@cshs.org (M.Q.); fqli2026@gmail.com (F.L.); yangmingjie@yahoo.com (M.Y.); lai.wang@cshs.org (L.W.); predimankrishan.shah@csmc.edu (P.K.S.); 2 Smidt Heart Institute and Department of Cardiology, Cedar-Sinai Medical Center, Los Angeles, CA 90048, USA

**Keywords:** Gata3, obesity, adipocyte, inflammation, diet-inducible regulator, chemokine

## Abstract

Adipose tissue dysfunction drives obesity-associated metabolic disease, yet the transcriptional regulators of pathological adipose remodeling remain undefined. Here, we identify adipocyte *Gata3* as a diet-inducible regulator that is dispensable for basal adipogenesis but necessary and sufficient for diet-induced obesity and systemic metabolic dysfunction. Adipocyte-specific *Gata3* deletion redirects adipose expansion from hypertrophic to hyperplastic growth and improves glucose tolerance, insulin sensitivity, and adipose inflammation, whereas human GATA3 re-expression in adipocytes restores obesity and metabolic dysfunction, suggesting cross-species conservation and establishing both necessity and sufficiency for a causal driver. Multi-omic profiling, integrating proteomics, metabolomics, and single-cell mass cytometry, reveals that Gata3 loss suppresses a STAT3-anchored inflammatory myeloid program while enhancing mitochondrial oxidative capacity. Through further tracing of this adipocyte-intrinsic program to distal metabolic organs, systemic lipidomic and chemokine profiling identifies a two-pronged mechanism through which adipocyte Gata3 drives hepatic dysfunction: selective remodeling of lipoprotein-surface lipids and a coordinated CXCL5/CXCL2/CXCL9 chemokine axis. Collectively, these findings reposition Gata3 from a putative anti-adipogenic brake to a diet-inducible master regulator of pathological adipose remodeling and nominate adipocyte-targeted Gata3 inhibition as a strategy to uncouple adipose expansion from systemic metabolic disease.

## 1. Introduction

Adipose tissue and the liver evolved to buffer intermittent nutrient availability, not the chronic caloric excess characteristic of modern obesity. Under sustained nutrient pressure, adipose tissue transitions from an adaptive storage organ into a dysfunctional immune and endocrine organ that drives systemic disease, yet the transcriptional programs governing this transition remain poorly defined. Adipocyte differentiation itself is orchestrated by a hierarchical transcriptional network centered on *Pparγ* and modulated by factors that either permit or restrain adipocyte formation [1]. *Gata3* has been widely proposed as a key brake on this network [1,2], a model that has shaped thinking about how adipose expansion might be targeted therapeutically, yet one that has never been rigorously tested with adipocyte-specific genetic resolution in vivo.

*Gata3*, classically defined by its roles in immune cell differentiation and lineage specification [3], was first implicated as an anti-adipogenic regulator in 3T3-F442A preadipocytes, where it was shown to bind the *Pparγ* proximal promoter and inhibit its activity; subsequent studies extended this interpretation to 3T3-L1 cells and nonspecific in vivo perturbations [4,5,6]. On the surface, these observations seemed to settle the case for an anti-adipogenic paradigm. The underlying evidence, however, is constrained by three compounding limitations. First, preadipocyte cell lines incompletely recapitulate in vivo adipocyte biology [7,8]. Second, promoter occupancy establishes binding but not transcriptional directionality, particularly given current understanding of distal regulatory elements and three-dimensional chromatin architecture [9,10,11,12], and *Gata3* itself can act as either an activator or repressor depending on the chromatin landscape and cofactor availability [11,13]. Third, in vivo delivery strategies using lentiviral or liposomal vectors alter *Gata3* expression across all cell types within adipose depots [5,6,14], a critical confound given that non-adipocytes outnumber adipocytes several-fold per gram of tissue [15] and that *Gata3* is functionally expressed across multiple stromal and immune lineages [16,17,18,19,20,21,22]. Together, these limitations mean that no existing study can definitively separate adipocyte-intrinsic Gata3 function from microenvironmental effects mediated by neighboring stromal or immune cells.

This distinction is not merely theoretical, it is precedented. We previously showed that macrophage-specific *Gata3* deficiency skews polarization toward an M2-like phenotype and improves cardiac function after myocardial infarction [22], directly demonstrating that *Gata3* activity in a non-adipocyte compartment can independently shape tissue-level outcomes and confound interpretations drawn from whole-tissue or nonspecific perturbations. This precedent motivated the present study: if *Gata3* function is cell-type-specific in the heart, its purported role as an adipogenic repressor, inferred largely from non-adipocyte-restricted models, warrants re-examination using tools with genuine cell-autonomous resolution.

Using adipocyte-restricted genetic models, we find not confirmation but a conceptual inversion of the prevailing model: *Gata3* is dispensable for basal adipogenesis, yet becomes selectively required for pathological adipose expansion under nutritional stress. *Gata3* is therefore not a constitutive repressor of adipocyte formation, but a context-dependent, diet-inducible regulator whose cell-autonomous activity in mature adipocytes is both necessary and sufficient to couple chronic nutrient excess to systemic metabolic disease. This reframing shifts *Gata3* from a passive brake on adipogenesis to an active, inducible driver of pathological remodeling, a distinction with direct translational implications.

## 2. Methods

### 2.1. Study Design

This study was designed to define the cell-autonomous role of *Gata3* in mature adipocytes during diet-induced obesity and to determine whether *Gata3* is necessary, sufficient, or merely associative for pathological adipose remodeling. We generated two complementary adipocyte-specific mouse models, an adipocyte-specific *Gata3* knockout (a*Gata3*KO) and an adipocyte-specific humanized *GATA3* knock-in (a*GATA3*KI), each evaluated under chow and high-fat diet (HFD) conditions. Unless indicated, experiments used male mice maintained on chow or HFD for up to 16 weeks. Individual animals were the experimental unit for in vivo, proteomic, metabolomic, lipidomic, and cytokine analyses; for CyTOF, stromal vascular fractions from three mice per group were pooled. Sample sizes were determined a priori from published variance estimates for metabolic phenotyping (80% power, α = 0.05). Investigators were blinded during tissue processing, histology, image quantification, and primary analysis; no animals or datasets were excluded. All procedures were approved by the Institutional Animal Care and Use Committee and performed per the NIH Guide for the Care and Use of Laboratory Animals.

### 2.2. Mouse Models

a*Gata3*KO mice were generated by crossing homozygous *Gata3*-floxed mice (loxP sites flanking exons 4 and 5) with Adipoq-Cre transgenic mice (Jackson Laboratory, Bar Harbor, ME USA 04609, stock no. 010803), which recombine selectively in terminally differentiated adipocytes. Recombination efficiency and specificity were verified by RT-PCR of fractionated adipose tissue; *Gata3* deletion was efficient in mature adipocytes but absent in the stromal vascular fraction. A constitutive rather than tamoxifen-inducible Cre strategy was used because tamoxifen independently alters adipose inflammation, glucose homeostasis, and systemic metabolism [23,24,25,26,27]. To test sufficiency and cross-species conservation, a*GATA3*KI mice were generated by crossing ROSA26-STOP-h*GATA3*-IRES-GFP mice (gift from M. Bouchard, McGill University [28]) with a*Gata3*KO mice, simultaneously deleting murine *Gata3* and activating human *GATA3* in mature adipocytes; species-specific RT-PCR confirmed correct, mutually exclusive transgene expression.

### 2.3. Dietary Challenge and Metabolic Phenotyping

Mice received standard chow (5% kcal fat) or HFD (45% kcal fat; Inotiv TD88137) from 6 to 8 weeks of age for 16–20 weeks, with weekly body-weight measurement. Food intake was measured over 10 days beginning 2 weeks post-HFD. Glucose tolerance tests (2 g/kg i.p. glucose, 16-h fast) and insulin tolerance tests (0.75 U/kg i.p. insulin, 6-h fast) were performed in chow-fed mice and repeated after 16 weeks of HFD; glucose excursion was quantified as area under the curve. At endpoint, adipose depots, liver, heart, and kidneys were dissected and weighed. Serum cholesterol, leptin, and adiponectin were measured by colorimetric assay and ELISA.

### 2.4. Histology, Cell Isolation, and Gene Expression

Adipose tissue was fixed, paraffin-embedded, and stained with hematoxylin and eosin; adipocyte size and number were quantified from ≥300 cells per animal using Fiji. Stromal vascular fraction cells were isolated by collagenase/dispase digestion and induced to differentiate in vitro using insulin, dexamethasone, and IBMX; lipid accumulation was assessed by Oil Red O staining and quantified by absorbance at 492 nm. Mature-adipocyte and stromal vascular fractions were separated by flotation to confirm recombination specificity. RNA was extracted with TRIzol and column purification; cDNA was synthesized and analyzed by quantitative PCR (SsoFast EvaGreen), normalized to Gapdh (2^−ΔΔCt^ method). Primer sequences are provided in the Supplementary Methods.

### 2.5. Proteomics, Metabolomics, CyTOF, Lipidomics, and Cytokine Profiling

Epididymal white adipose tissue proteomes (n = 5/group) were profiled by data-independent acquisition LC-MS/MS (Orbitrap Astral) following protein-aggregation-capture sample preparation [20]; raw data were processed in DIA-NN [21] and analyzed in FragPipe-Analyst using limma [22], with differential abundance defined as adjusted *p* ≤ 0.05 and |log_2_FC| ≥ 1. Targeted adipose metabolomics (n = 3/group) used dynamic multiple-reaction-monitoring LC-MS/MS (Agilent 6470A) covering 219 polar metabolites, analyzed in MetaboAnalyst 5.0. Single-cell mass cytometry (CyTOF) was performed on pooled stromal vascular fraction cells from inguinal and gonadal adipose tissue (3 mice/pool) using a 33-metal-isotope antibody panel and a Helios mass cytometer, essentially as described [23]. Serum lipidomics used differential-mobility-spectrometry shotgun lipidomics (SCIEX Triple Quad 6500+, SCIEX, Framingham, MA, USA) covering approximately 1450 species across 17 subclasses, normalized to isotopically labeled internal standards [24].Circulating cytokines and chemokines were quantified using the MILLIPLEX® MAP Mouse Cytokine/Chemokine Magnetic Bead Panel (32-plex; MilliporeSigma, Burlington, MA, USA). Full instrument parameters, antibody panels, and reagent sources are provided in the Supplementary Methods.

### 2.6. Statistical Analysis

Analyses used GraphPad Prism 11 unless noted. Data are mean ± s.e.m. Normality was assessed by Shapiro–Wilk testing; statistical significance was assessed using Welch’s corrected two-tailed *t*-test for two-group comparisons, and longitudinal or multi-group data used two-way repeated-measures ANOVA with Tukey’s correction. High-dimensional datasets were analyzed with dataset-specific pipelines (limma with Benjamini–Hochberg correction for proteomics; two-proportion z-tests with FDR correction for CyTOF), as detailed above and in the Supplementary Methods. Significance was defined as *p* < 0.05 (* *p* < 0.05, ** *p* < 0.01, *** *p* < 0.001, **** *p* < 0.0001).

### 2.7. Data Availability

Proteomic, metabolomic, lipidomic, multiplex cytokine, and CyTOF datasets will be deposited in publicly accessible repositories before publication; accession numbers will be provided upon acceptance. The source data underlying all figures will be included with the Supplementary Information upon acceptance.

## 3. Results

### 3.1. Gata3 Expression Is Depot-Selective and Nutritionally Regulated, and Its Co-Ordination with Pparγ Argues Against Obligate Antagonism

**Figure 1:**
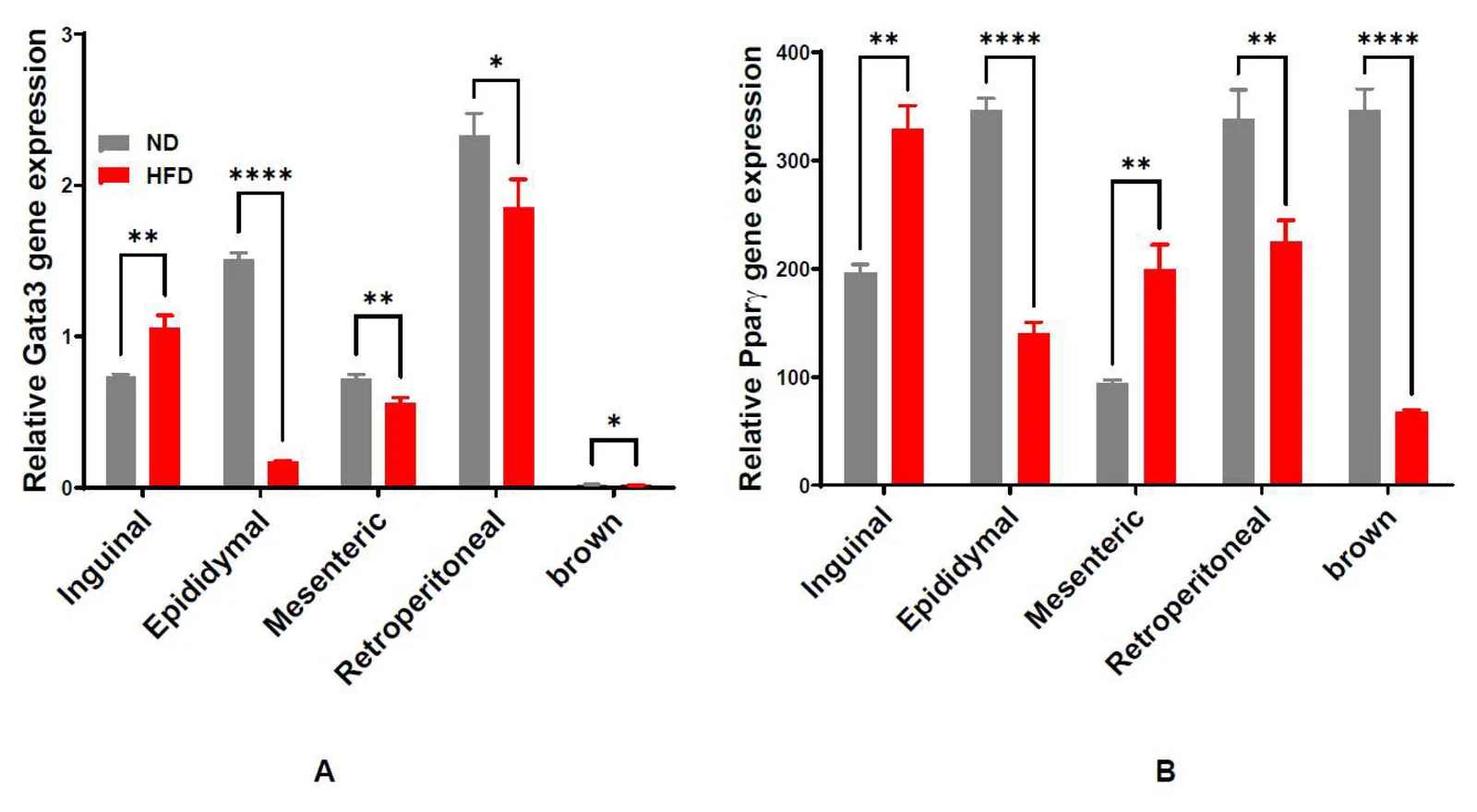
Gata3 expression is depot-specific and regulated by nutritional status. Relative *Gata3* mRNA expression across major adipose depots from chow-fed, designated as ND, and HFD-fed mice. qPCR was performed on total RNA from adipose tissue (n = 4–6 mice per condition) and normalized to *Gapdh* (glyceraldehyde 3-phosphate dehydrogenase). (**A**), *Gata3*. (**B**), *Pparγ*. Statistical significance was assessed using Welch’s corrected two-tailed *t*-test comparing ND versus HFD (GraphPad Prism v11.0.0).

Adipose depots differ markedly in developmental origin, metabolic function, and susceptibility to obesity-associated dysfunction, yet whether transcriptional regulators implicated in adipose pathology are uniformly deployed or exhibit depot-specific control remains unclear. To address this, we systematically mapped basal and nutritionally regulated *Gata3* expression across major adipose depots. Under chow-fed conditions, *Gata3* transcripts were detected in all depots examined but exhibited a graded hierarchy: expression was highest in visceral compartments, particularly retroperitoneal and epididymal adipose tissue, and substantially lower in inguinal and mesenteric fat (Figure 1A). *Pparγ* displayed a remarkably concordant distribution, with preferential enrichment in epididymal and retroperitoneal depots (Figure 1B). The parallel depot-selective enrichment of both factors under basal conditions argues against their characterization as obligate transcriptional antagonists, and instead indicates that *Gata3* and *Pparγ* occupy a shared transcriptional context in mature adipocytes, a co-regulatory relationship whose nutritional dynamics we examine next.

HFD feeding produced depot-selective transcriptional remodeling rather than a uniform amplification of the basal pattern. *Gata3* mRNA declined in most visceral depots while remaining stable or modestly increasing in inguinal adipose tissue (Figure 1A); *Pparγ* closely mirrored these changes under the same dietary challenge (Figure 1B). The coordinated, depot-selective regulation of both factors across basal and obesogenic states positions *Gata3* as a context-dependent transcriptional mediator jointly shaped by anatomical depot identity and nutritional state, a conclusion that requires careful interpretation of the normalization strategy underlying the expression data.

The apparent decline in visceral *Gata3* and *Pparγ* expression under HFD does not reflect a genuine transcriptional repression of these genes in adipocytes, but is instead a cell-composition confound arising from whole-tissue GAPDH normalization. Under HFD, visceral adipose tissue undergoes marked inflammatory infiltration: immune cells, which express GAPDH at high levels but contribute negligible adipocyte signal, expand substantially and dilute the adipocyte-derived mRNA signature. Concurrently, pathological visceral fat expansion is driven predominantly by adipocyte hypertrophy: existing adipocytes enlarge without a proportional increase in cell number, so adipocyte-derived GAPDH per milligram of tissue remains approximately constant even as total depot GAPDH rises sharply with immune cell influx. Normalizing a stable adipocyte *Gata3* signal to this expanded denominator therefore arithmetically suppresses its apparent expression, not because adipocyte transcription is genuinely reduced, but because the adipocyte-derived signal is progressively diluted by a non-adipocyte GAPDH pool that grows in proportion to the inflammatory burden.

This cell-composition interpretation is directly confirmed by the inguinal adipose tissue data (Figure 1A,B). The inguinal subcutaneous depot sustains substantially less HFD-induced inflammatory infiltration than visceral depots, an established depot-specific difference, and correspondingly shows stable or increased, rather than decreased, *Gata3* and *Pparγ* expression under HFD. This is the precise directional pattern the cell-composition confound predicts: a depot with minimal immune expansion preserves its GAPDH denominator and thus its apparent adipocyte marker expression, whereas a depot with extensive immune expansion suppresses it. The apparent HFD expression data therefore report the magnitude of adipose tissue inflammatory pathology rather than the intrinsic transcriptional response of adipocytes to diet, a distinction that reinforces, rather than undermines, the central conclusion of this study.

**Figure 2:**
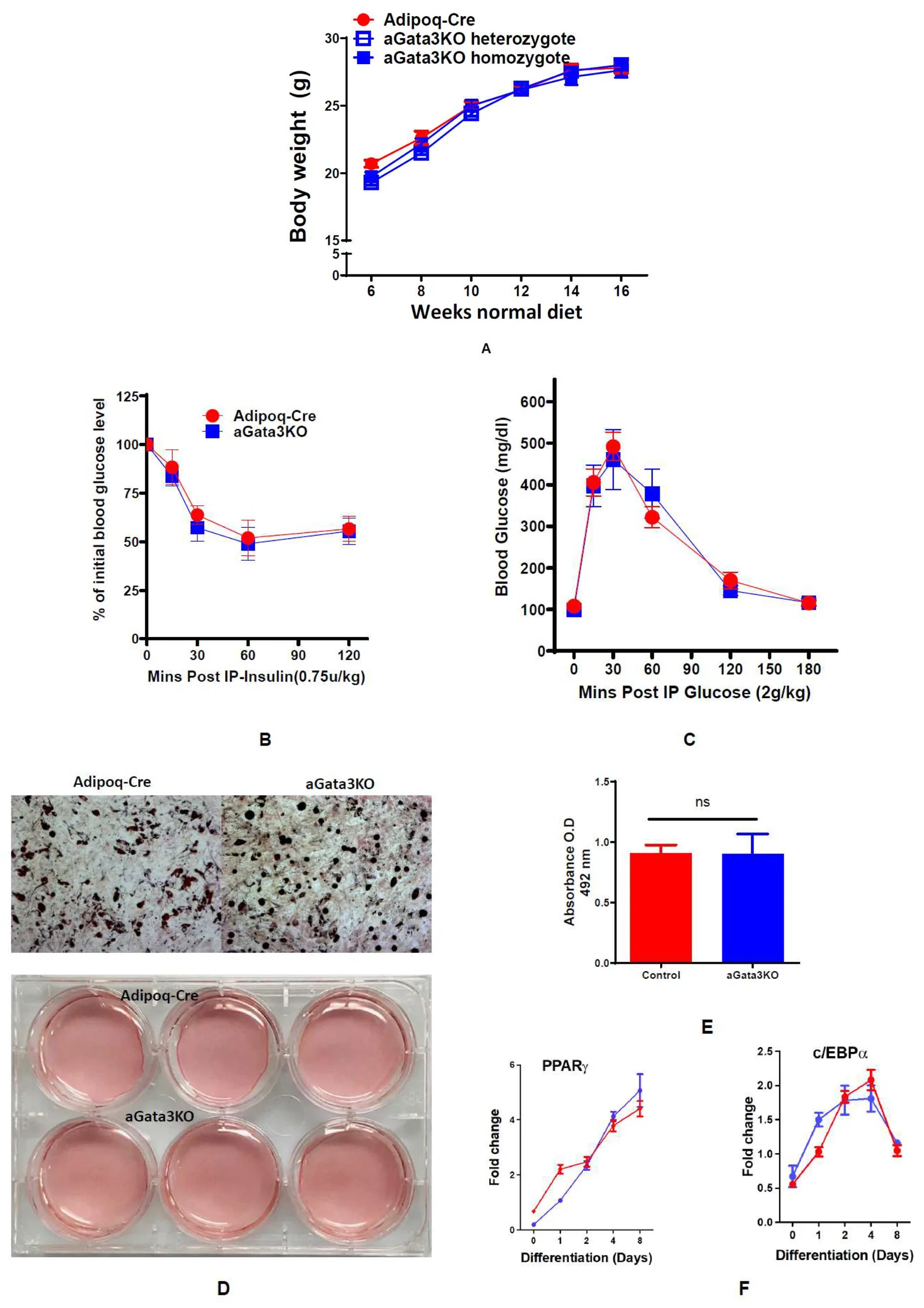
Adipocyte-specific deletion of Gata3 does not affect adipogenesis or metabolic homeostasis under basal conditions. (**A**), Body weight of Adipoq-Cre controls and heterozygous and homozygous adipocyte-specific *Gata3* knockout (aGata3KO) mice on chow diet (n = 9–12 mice per genotype). (**B**,**C**), Glucose tolerance and insulin tolerance tests showing comparable glucose handling and insulin sensitivity among genotypes (n = 5–7 mice per genotype). (**D**), Representative Oil Red O staining of SVF cells isolated from epididymal fat of the indicated genotypes after 16 weeks of HFD and differentiated ex vivo with adipogenic cocktail (IBMX, 0.5 mM; dexamethasone, 1 μM; insulin, 10 μg mL^−1^). (**E**), Quantification of lipid accumulation in differentiated adipocytes by dye extraction and absorbance at 492 nm. (**F**), qPCR analysis of adipogenic markers, including *Pparγ* and *Cebpα*, during differentiation. Total RNA was isolated from SVF-derived adipocytes at the indicated time points. Data are representative of three independent experiments from distinct cell isolates. Statistical significance was assessed using Welch’s corrected two-tailed *t*-test (GraphPad Prism v11.0.0). Statistically insignificant numbers are designated as ns. Red represents control groups and blue represents aGata3KO mice.

To determine the cell-autonomous consequences of adipocyte *Gata3* loss, we generated adipocyte-specific *Gata3* knockout mice (aGata3KO) by crossing *Gata3*-floxed mice with Adipoq-Cre transgenic mice. Because Adipoq-Cre selectively targets terminally differentiated adipocytes while sparing preadipocyte progenitor populations [29,30], this model permits evaluation of mature-adipocyte *Gata3* function independently of developmental confounders. Under chow-fed conditions, heterozygous and homozygous aGata3KO mice were indistinguishable from Adipoq-Cre controls at study initiation (19.74 g versus 20.70 g; Figure 2A), gained weight comparably (~29 g by 16 weeks, ~45% increase), and maintained equivalent glucose tolerance and insulin sensitivity across genotypes (Figure 2B,C).

To test whether *Gata3* influences adipogenic capacity at the cell-intrinsic level, stromal vascular fraction (SVF) cells isolated from both genotypes were induced to differentiate ex vivo. Morphological assessment revealed no differences in lipid droplet accumulation (Figure 2D), quantitative lipid extraction confirmed equivalent ex vivo lipid storage (Figure 2E), and expression of *Pparγ* and *C/ebpα* remained unchanged throughout the differentiation time course (Figure 2F). These in vivo and ex vivo data converge on a single conclusion: *Gata3* is entirely dispensable for adipocyte differentiation, lipid storage, and the maintenance of systemic metabolic homeostasis under physiological nutritional conditions. The depot-specific and nutritionally regulated *Gata3* expression pattern we characterize above therefore reflects an adaptive transcriptional response in mature adipocytes, not a developmental limitation, and provides the mechanistic backdrop for its diet-inducible role in pathological adipose remodeling.

### 3.2. Adipocyte Gata3 Drives Diet-Induced Obesity and Metabolic Dysfunction

**Figure 3:**
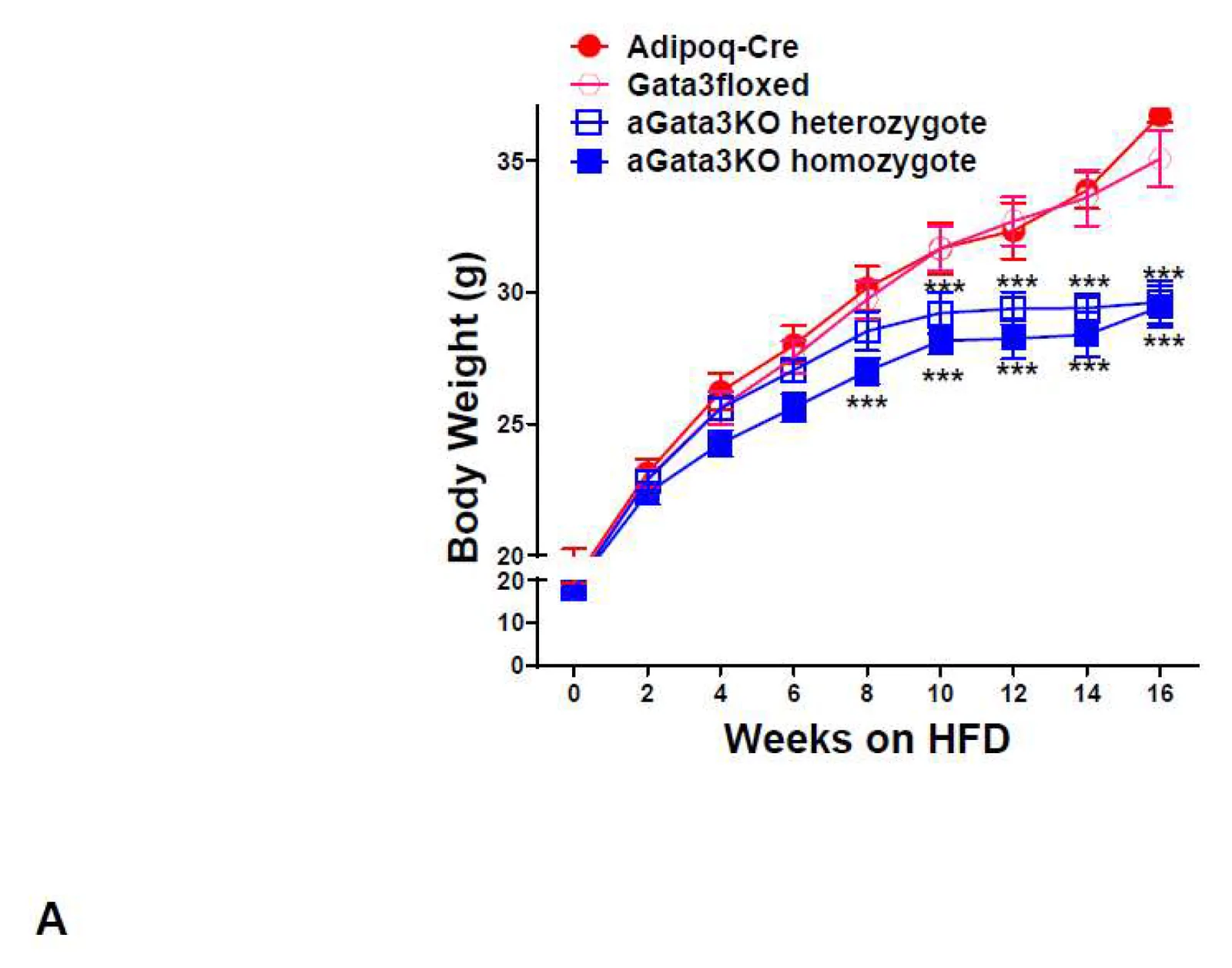

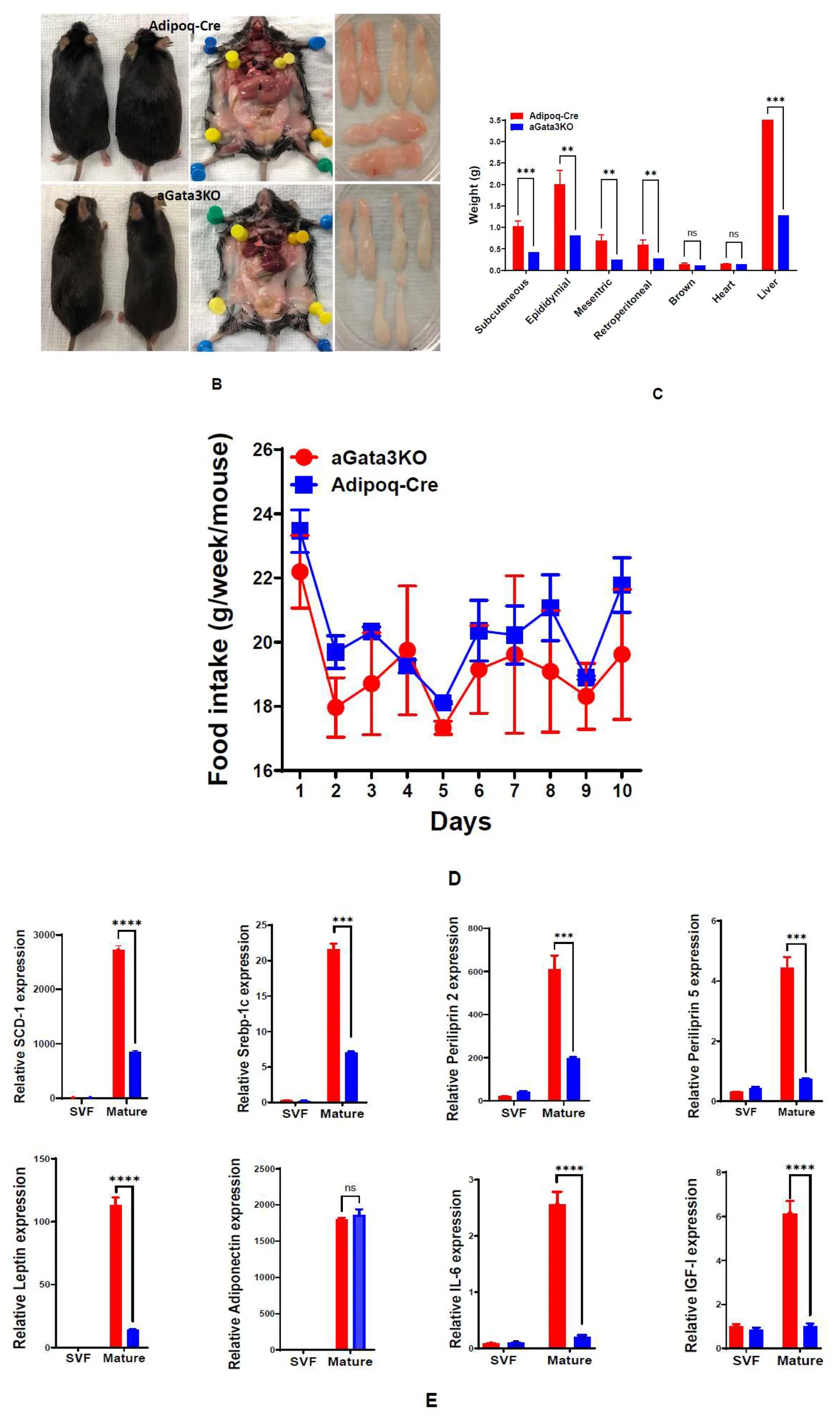

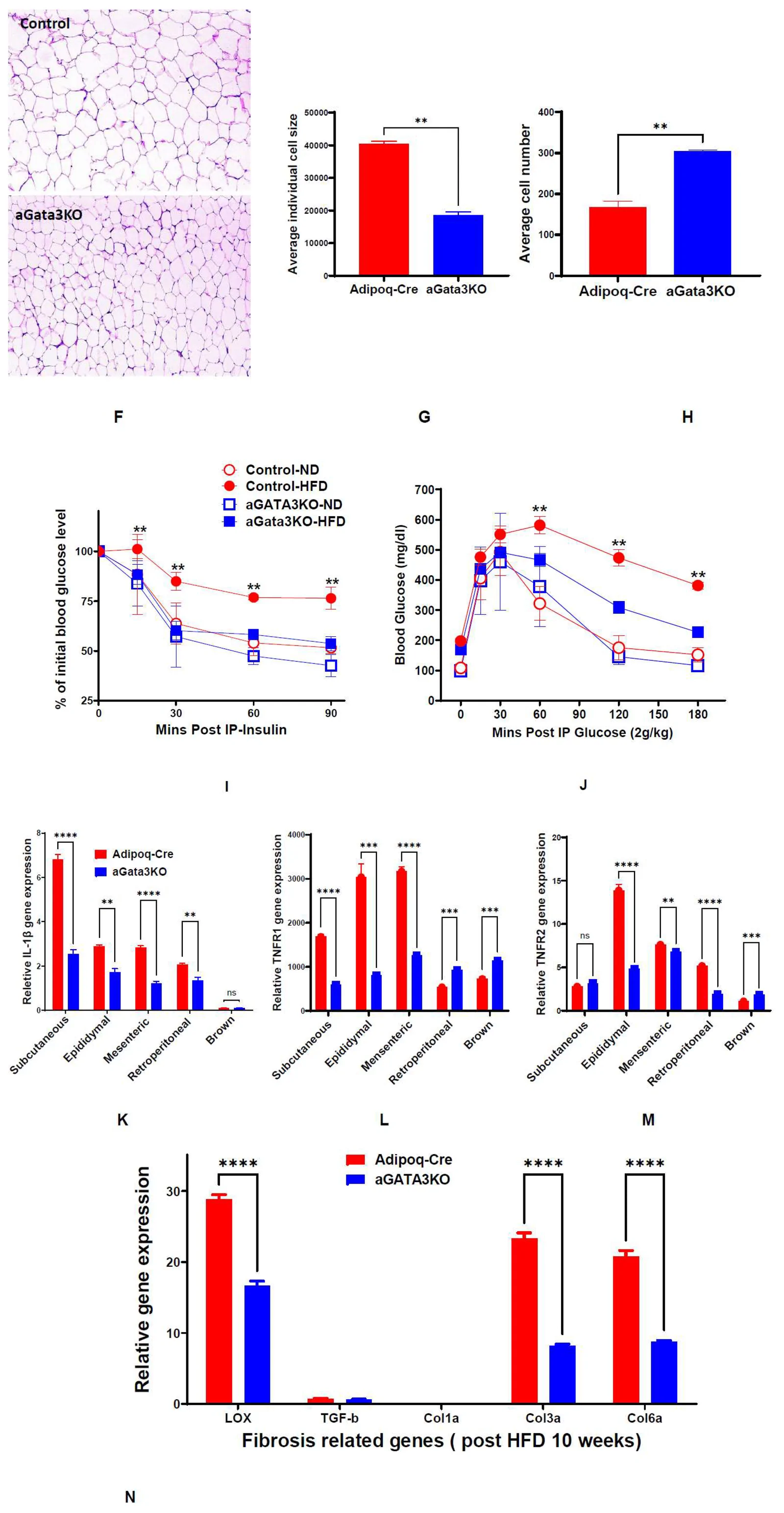
Adipocyte-specific Gata3 deficiency protects against diet-induced obesity and metabolic dysfunction. (**A**), Body weight trajectories during HFD feeding (n = 12–15 mice per genotype). (**B**), Representative photographs of whole mice, dissected animals, and epididymal fat pads after 16 weeks of HFD. (**C**), Weights of adipose depots and selected organs following HFD. (**D**), Food intake measurements. (**E**), qPCR analysis of adipocyte markers in isolated mature adipocytes and stromal vascular fraction (SVF). (**F**), Representative H&E-stained sections of epididymal adipose tissue after 16 weeks of HFD. (**G**,**H**), Quantification of adipocyte size and number from histological sections; values represent mean cell area and cell number averaged across four non-overlapping fields per sample. (**I**,**J**), Glucose tolerance and insulin tolerance tests after HFD feeding (n = 5 mice per genotype). (**K**–**M**), qPCR analysis of inflammatory mediator expression in epididymal adipose tissue after 16 weeks of HFD. (**N**), qPCR analysis of fibrosis-associated genes in epididymal adipose tissue. Statistical significance was assessed by two-tailed *t*-test.

The dispensability of *Gata3* under chow-fed conditions raised the possibility that its function is specifically engaged under chronic caloric excess. To test this, we subjected aGata3KO and control mice to 16 weeks of high-fat diet (HFD). Control Adipoq-Cre and *Gata3*-floxed mice reached comparable body weights of 36.73 g and 35.78 g, approximately 25% above chow-fed levels (Figure 3A). In striking contrast, both heterozygous and homozygous aGata3KO mice remained markedly resistant to weight gain, plateauing at 29.62 g and 29.30 g. Divergence emerged by four weeks after HFD initiation and reached statistical significance by week eight, indicating that loss of adipocyte *Gata3* provides sustained protection against diet-induced obesity rather than a transient delay in weight gain.

Reduced body mass reflected selective attenuation of adipose accumulation rather than a generalized growth defect. All major white adipose depots, subcutaneous, epididymal, mesenteric, and retroperitoneal, were significantly reduced, whereas brown adipose tissue and heart weight were unchanged and liver weight was decreased, consistent with diminished ectopic lipid deposition (Figure 3B,C). Food intake did not differ between genotypes (Figure 3D), ruling out altered caloric consumption and localizing the protective effect to adipocyte-intrinsic *Gata3* function. Fractionation confirmed efficient, adipocyte-selective deletion: expression of canonical adipocyte genes (Scd1, Srebp1c, Perilipin2, Perilipin5, leptin, adiponectin, IL-6, and Igf1) was markedly reduced in mature adipocytes but unchanged in the stromal vascular fraction (Figure 3E). Despite this remodeling, adiponectin expression was preserved, indicating maintained adipocyte identity. Histological analysis revealed a fundamental shift in tissue architecture, with aGata3KO depots containing substantially smaller adipocytes and displaying ~50% reduced cell size and ~80% increased adipocyte number (*p* < 0.01; Figure 3F–H). This transition from hypertrophic to hyperplastic expansion is a defining feature of metabolically healthy adipose tissue and foreshadows the oxidative, anti-inflammatory signatures revealed by our multi-omic analyses.

These structural improvements translated into robust metabolic benefits. Although glucose tolerance and insulin sensitivity were comparable under chow feeding, both were significantly improved in aGata3KO mice following HFD (Figure 3I,J), demonstrating that the architectural remodeling is functionally consequential. Improved systemic parameters coincided with suppression of adipose inflammation, reflected by lower IL-1β, TNFR1, and TNFR2 expression (Figure 3K–M), and diminished fibrogenic gene expression (Figure 3N). Together with the proteomic, metabolomic, and CyTOF data presented below, these findings establish adipocyte *Gata3* as a pathogenic driver that couples nutrient excess to hypertrophic adipose expansion, inflammatory myeloid programming, and systemic metabolic deterioration. In doing so, they directly overturn the prevailing view of *Gata3* as a constitutive anti-adipogenic regulator and instead position it as a diet-inducible, adipocyte-intrinsic effector whose inhibition preserves both adipose function and multi-organ metabolic health under chronic caloric load.

### 3.3. Adipocyte Gata3 Is Sufficient to Drive Diet-Induced Obesity and Is Conserved Between Species

**Figure 4:**
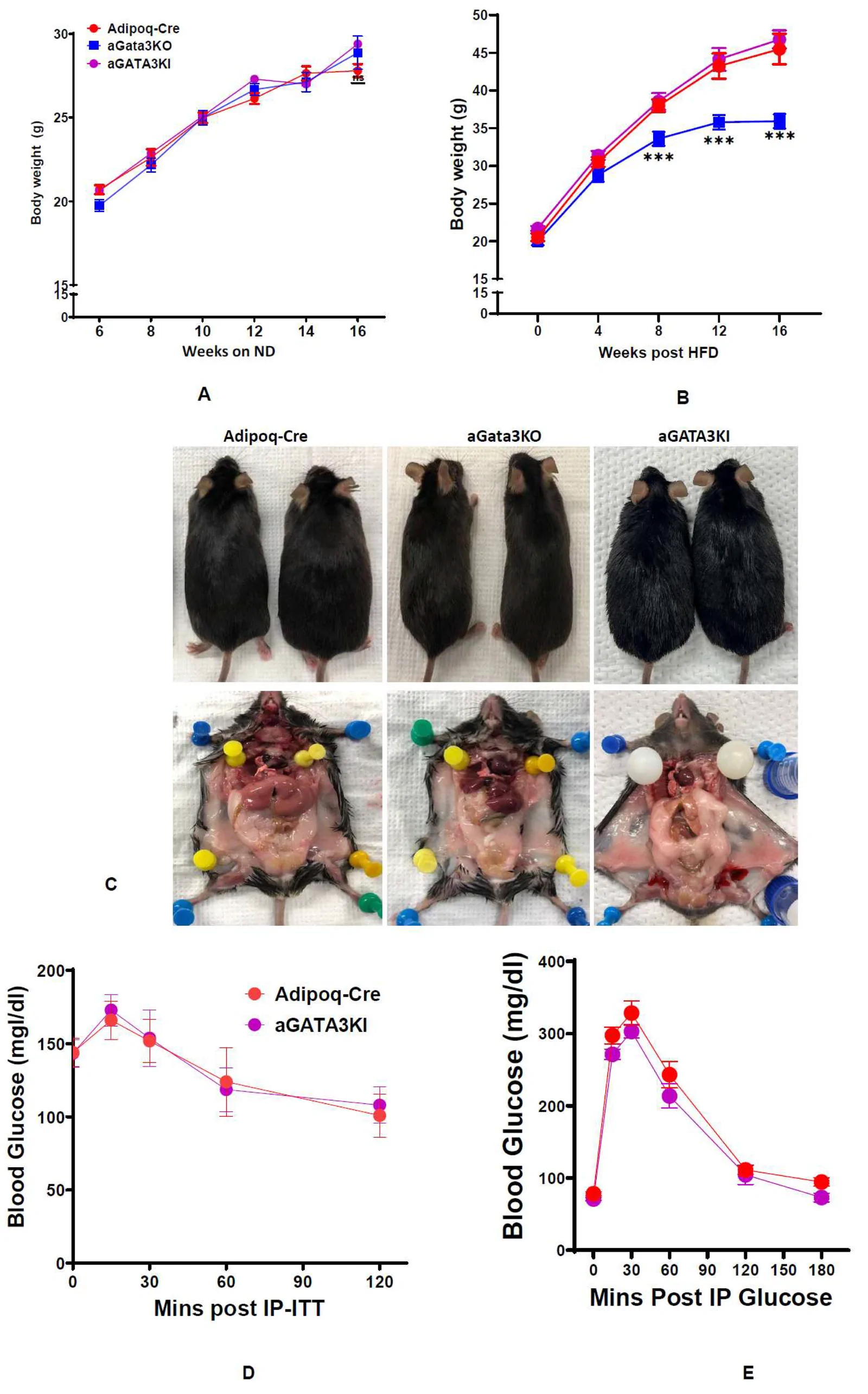

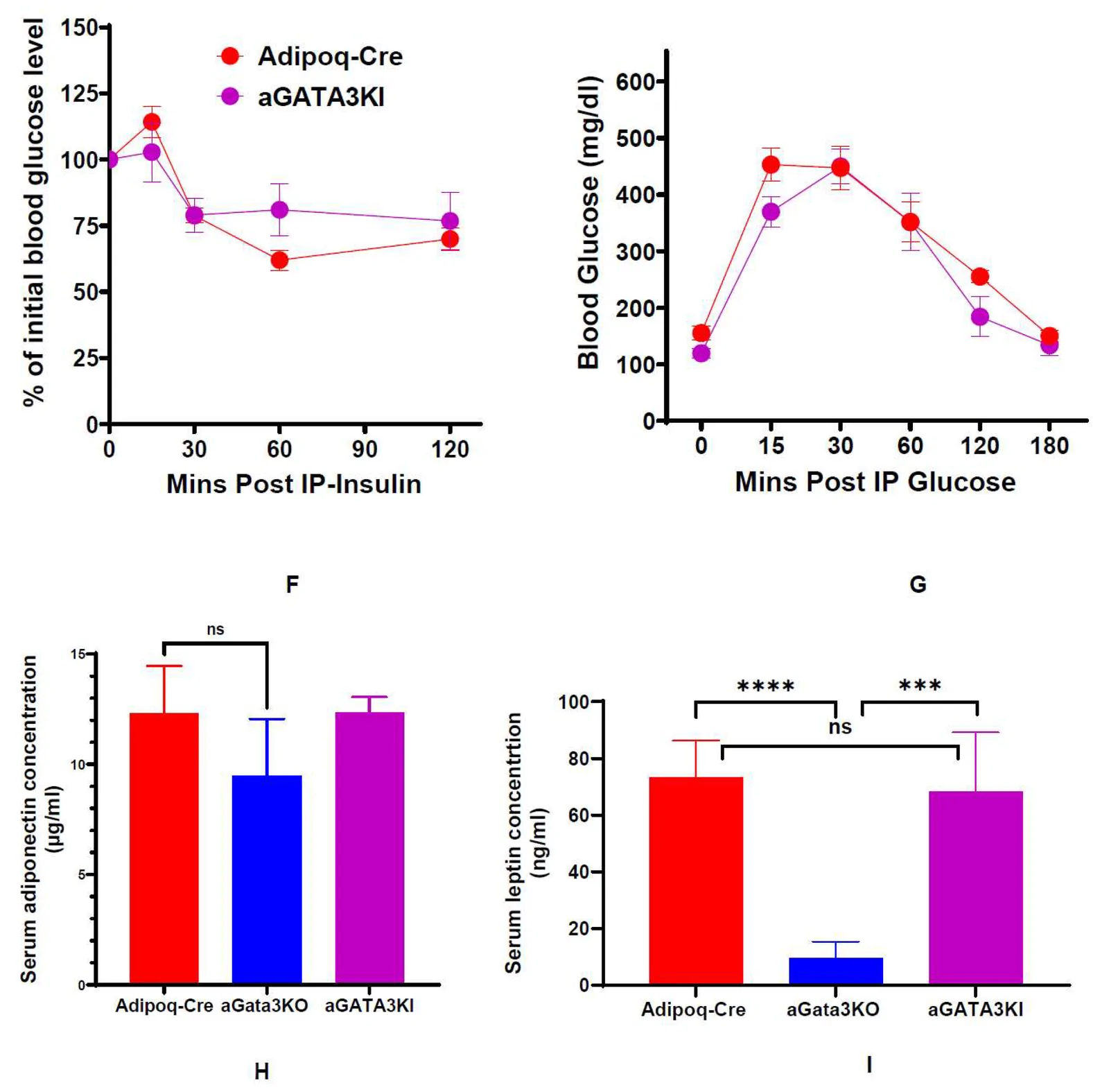
Adipocyte-specific expression of human GATA3 restores obesity-associated phenotypes in Gata3-deficient mice. (**A**,**B**) Body-weight trajectories of the indicated genotypes during chow (ND) feeding (n = 8–12 mice per genotype) and high-fat diet (HFD) feeding (n = 12–15 mice per genotype). (**C**) Representative images of mice from each genotype and their corresponding dissected adipose depots. (**D**–**G**) Glucose tolerance tests and insulin tolerance tests performed in mice maintained on chow diet or HFD (n = 5 mice per genotype). (**H**,**I**) Circulating adiponectin and leptin concentrations, measured by ELISA (n = 4 mice per genotype).

Having established that adipocyte *Gata3* is necessary for diet-induced obesity, we next asked whether its expression is also sufficient, and whether this function is conserved in the human ortholog. To address this, we generated an adipocyte-specific humanized knock-in model (aGATA3KI) in which human *GATA3* is re-expressed in adipocytes on the aGata3KO background. At study initiation, body weights were comparable across Adipoq-Cre controls, aGata3KO, and aGATA3KI mice (~20 g; Figure 4A), and all groups gained weight equivalently under chow feeding, reaching ~30 g after 16 weeks (~50% increase; Figure 4A). The absence of any basal phenotype confirms that human *GATA3* re-expression does not perturb normal growth or energy balance, ensuring that any HFD-dependent effects can be attributed specifically to the obesogenic function of *Gata3*.

Under HFD, striking genotype-dependent divergence emerged. Consistent with prior observations, aGata3KO mice remained relatively lean (~35 g). By contrast, aGATA3KI mice gained weight indistinguishably from controls, reaching ~45 g after 16 weeks, approximately 28% heavier than aGata3KO mice (Figure 4B). Depot-level analysis corroborated these findings, demonstrating substantial restoration of adiposity across major white adipose compartments in aGATA3KI mice relative to knockouts (Figure 4C). Thus, re-expression of human *GATA3* in mature adipocytes alone is sufficient to fully reconstitute diet-induced adipose expansion.

Metabolic phenotyping showed that human *GATA3* re-expression restored not only adiposity but the full spectrum of obesity-associated metabolic dysfunction. Glucose tolerance and insulin sensitivity were indistinguishable between aGATA3KI mice and controls under both chow and HFD conditions (Figure 4D–G), establishing that the protection conferred by *Gata3* deletion is completely reversed by adipocyte-restricted human *GATA3* re-expression. Circulating adiponectin remained comparable across all three genotypes under HFD (Figure 4H), indicating preserved adipocyte identity and endocrine function. In contrast, circulating leptin closely tracked adipose mass, reaching ~75 ng/mL in both control and aGATA3KI mice but only ~10 ng/mL in aGata3KO animals (Figure 4I), a sevenfold difference that reflects the magnitude of adipose expansion restored by human *GATA3*.

Collectively, these data demonstrate that adipocyte *Gata3*/*GATA3* is both necessary and sufficient for diet-induced adipose expansion and metabolic dysfunction, operates cell-autonomously, and is functionally conserved between murine and human orthologs. This cross-species conservation strongly supports *GATA3* as a translational target in human obesity and provides a genetic framework for selectively modulating adipocyte-intrinsic pathogenic programs without disrupting basal adipocyte identity.

### 3.4. Global Proteomic Profiling Identifies Gata3 as a Key Regulator of Inflammatory and Cytoskeletal Remodeling in Obese Adipose Tissue

**Figure 5:**
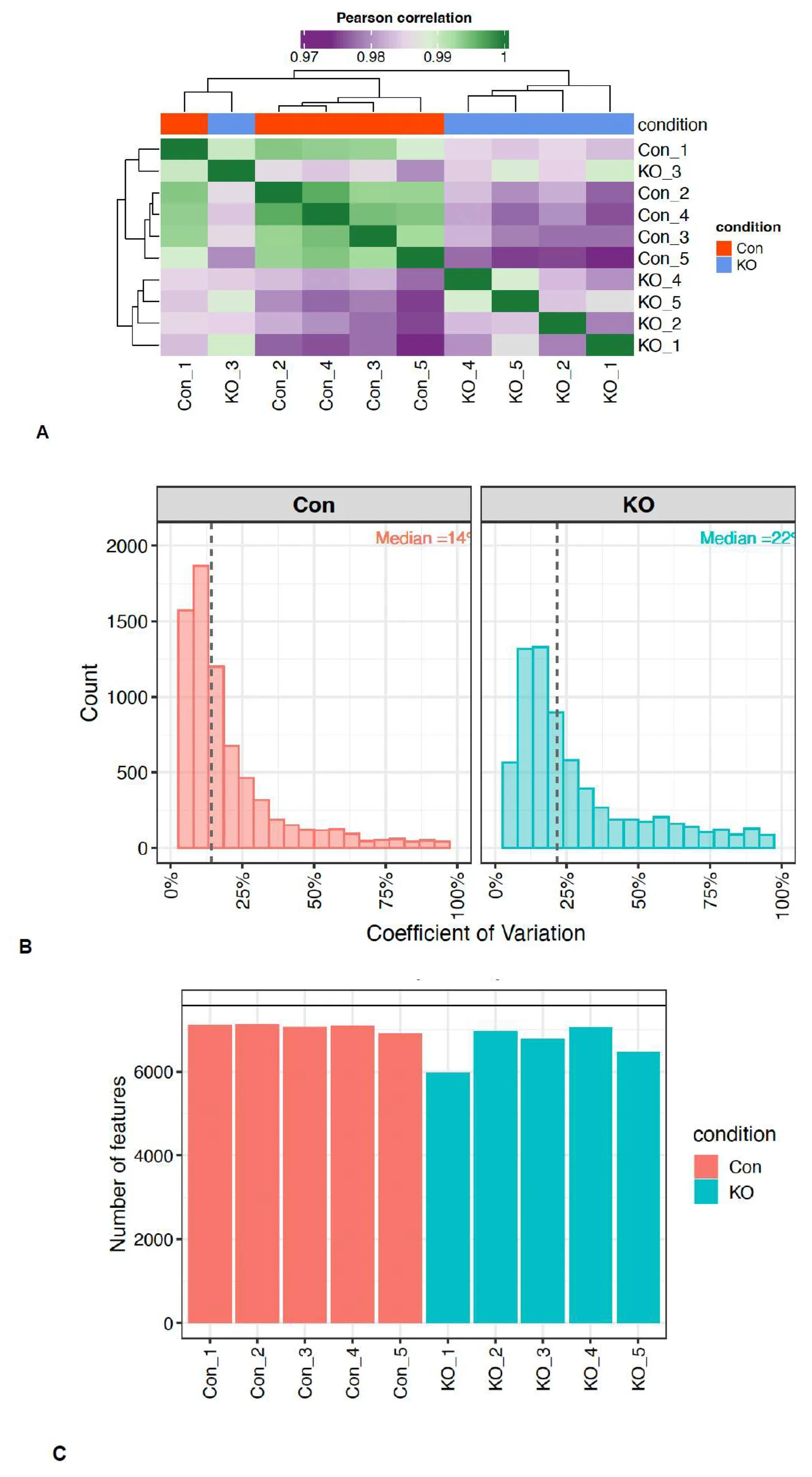

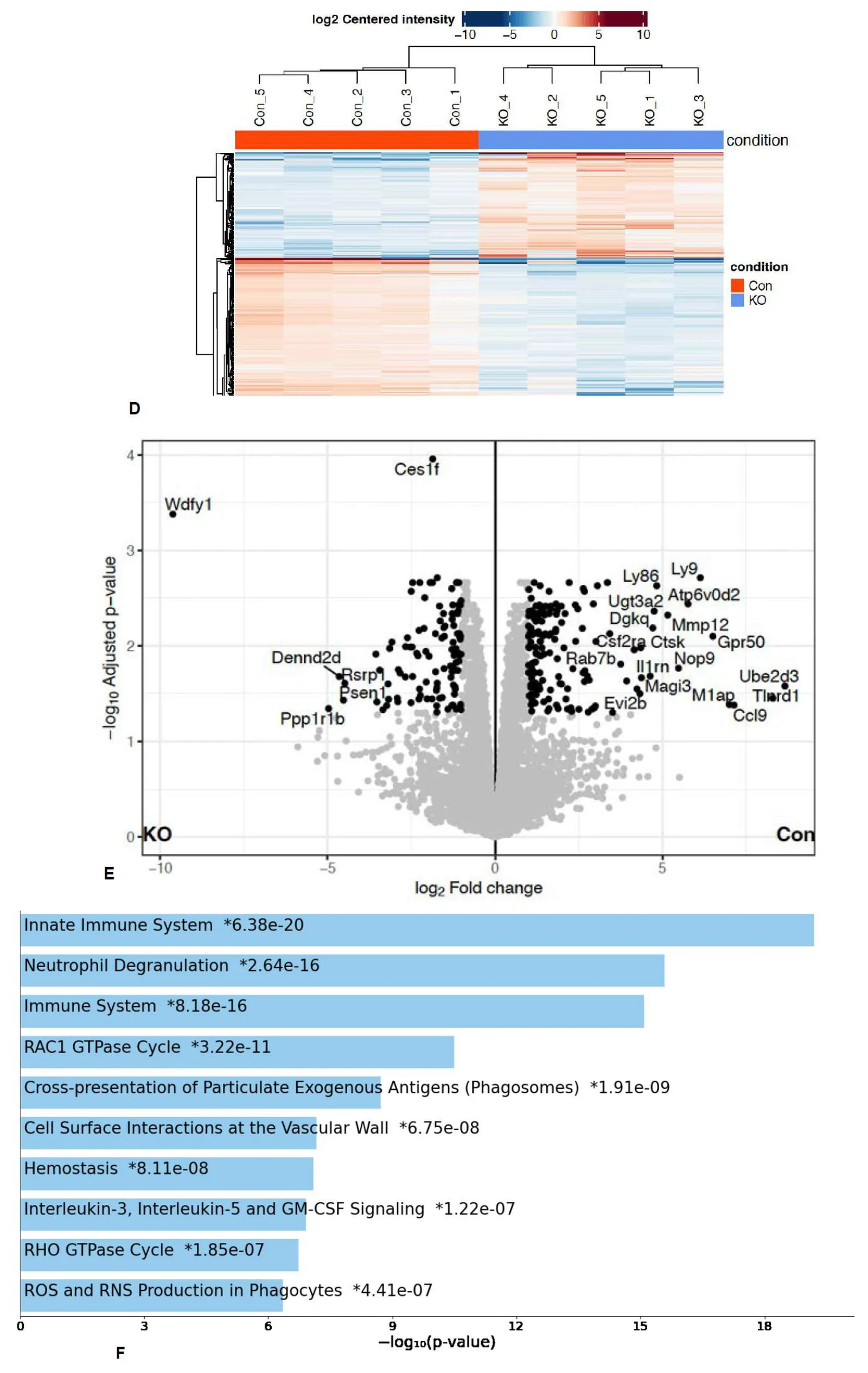

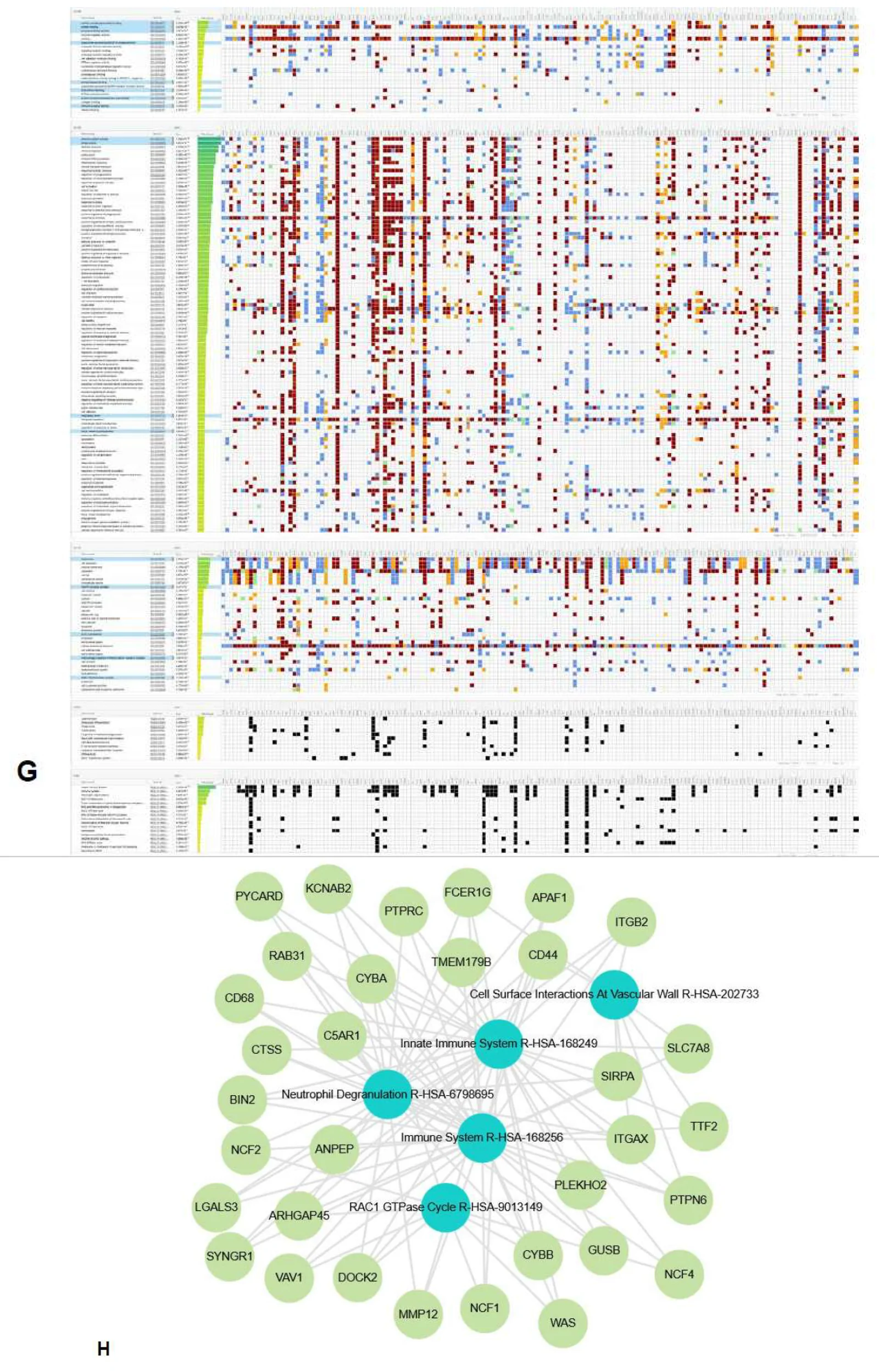

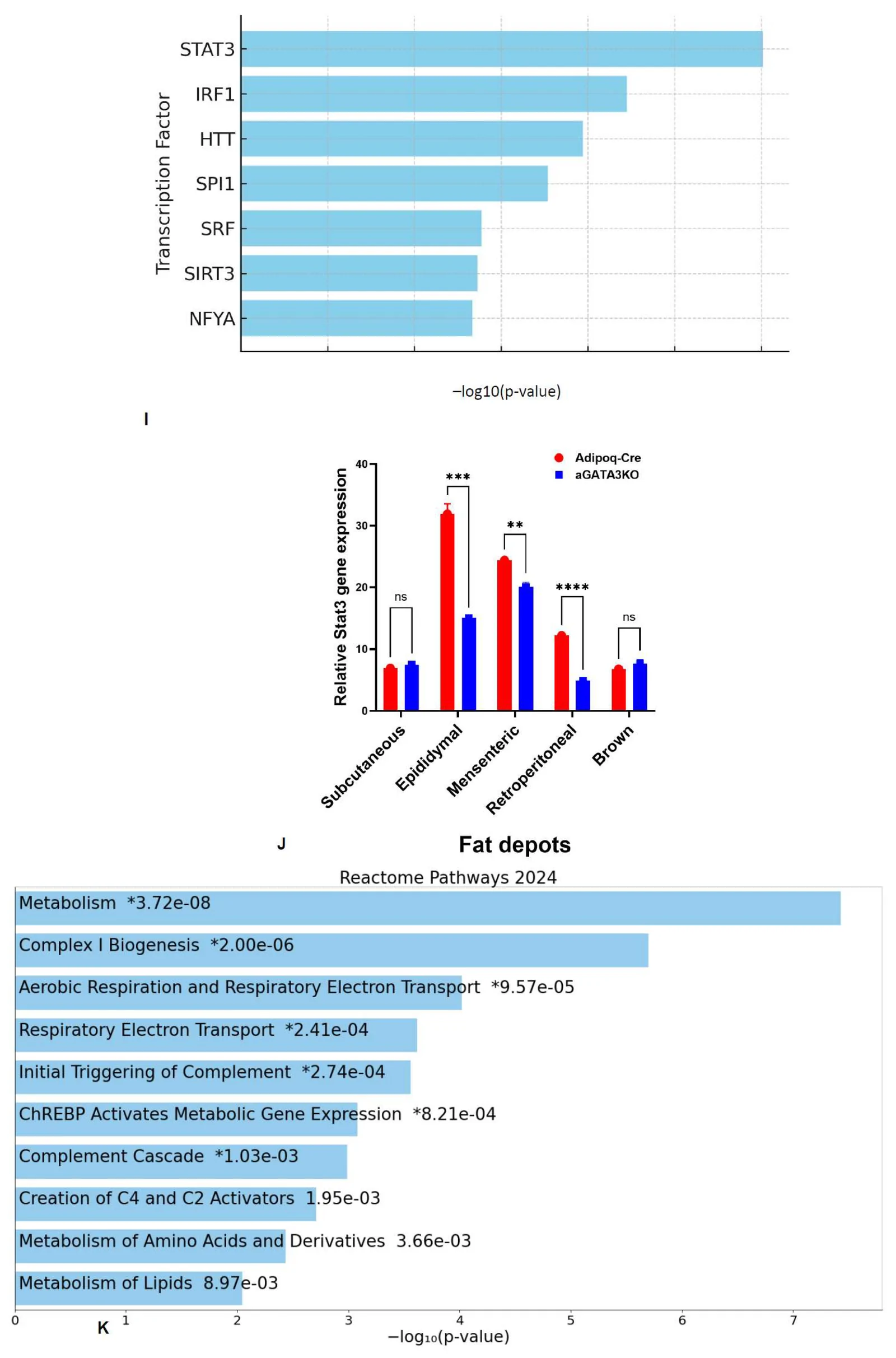

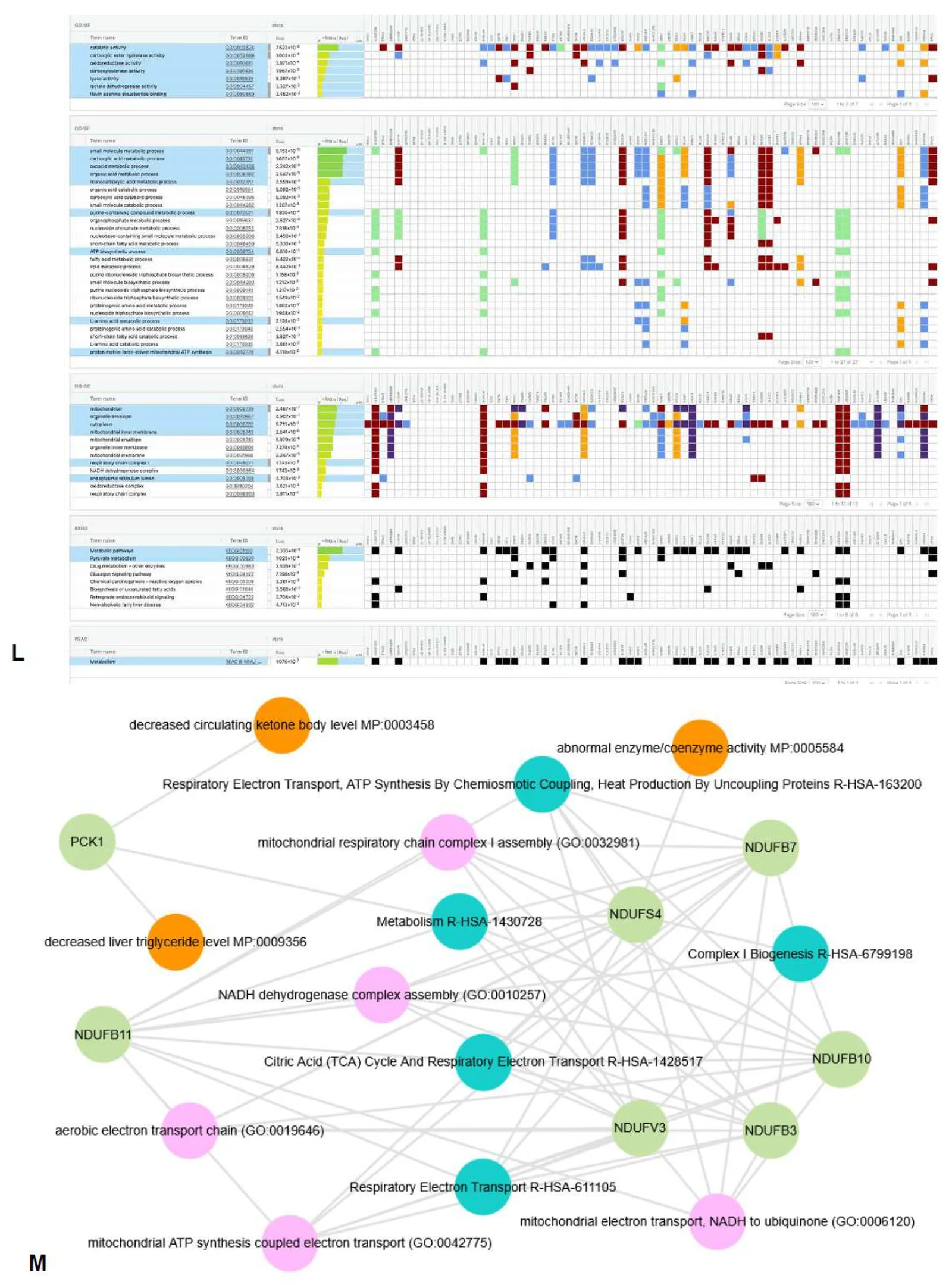
Global proteomic profiling identifies inflammatory and metabolic pathways regulated by adipocyte Gata3. (**A**) Pearson correlation matrix of epididymal white adipose tissue (eWAT) proteomes from high-fat diet (HFD)-fed Adipoq-Cre control mice (Con; n = 5) and adipocyte-specific Gata3 knockout mice (aGata3KO; KO; n = 5). (**B**,**C**) Proteomic quality-control metrics, including coefficients of variation and proteome coverage. (**D**) Heat map of differentially abundant proteins in eWAT from control and aGata3KO mice after 16 weeks of HFD feeding. (**E**) Volcano plot showing proteins differentially abundant following adipocyte-specific Gata3 deletion. (**F**,**G**) Enrichment analyses of protein classes and associated functional pathways among proteins enriched in control eWAT, including Reactome pathway annotations. (**H**) Protein–protein interaction network and hub-protein analysis of the control-enriched protein module. (**I**) Transcription-factor enrichment analysis of proteins enriched in control mice, highlighting STAT3-associated inflammatory signaling. (**J**) Quantitative PCR analysis of *Stat3* mRNA expression in the indicated adipose depots after 16 weeks of HFD feeding, showing reduced *Stat3* expression in epididymal, mesenteric, and retroperitoneal adipose tissue from aGata3KO mice. (**K**,**L**) Enrichment analyses of protein classes and functional pathways among proteins enriched in aGata3KO eWAT. (**M**) Network-topology analysis of metabolically enriched protein modules in aGata3KO adipose tissue. Functional enrichment and network analyses were performed using g:Profiler and Enrichr.

We next performed quantitative proteomic profiling of eWAT from HFD-fed control and aGata3KO mice, quantifying 7580 proteins. Hierarchical clustering of pairwise Pearson correlations achieved complete genotype segregation (r > 0.98; Figure 5A), confirming genotype as the dominant source of variance. Within-group coefficients of variation were well controlled (control: 14%; KO: 22%; Figure 5B), and proteome depth was comparable across samples (6800–7100 proteins per control; 6000–7000 per KO; Figure 5C), noteworthy given the dynamic range constraints imposed by abundant lipid droplet-associated proteins in adipose proteomics [31]. Differential expression analysis (FDR < 0.05; |log_2_FC| ≥ 1.0) identified a high-confidence set of remodeled proteins whose unsupervised clustering independently recapitulated genotype segregation (Figure 5D), indicating that these changes represent a robust biological signal rather than statistical noise. Effect sizes were substantial, with many proteins approaching |log_2_FC| ≥ 3, magnitudes that exceed typical single-gene knockout transcriptomic studies [32,33] and suggest that *Gata3* loss triggers proteome-wide reorganization rather than discrete, isolated changes.

The volcano plot revealed an additional dimension of biological asymmetry (Figure 5E). The control-enriched arm was not only wider but also more densely populated with statistically significant proteins and reached substantially higher −log_10_-adjusted *p*-values than the KO-enriched arm. Such a pronounced and directional imbalance is unlikely to reflect technical variance alone and instead points to a primary biological signal: the dominant axis of *Gata3*-dependent remodeling *is* active acquisition of an inflammatory proteome in *Gata3*-sufficient tissue, rather than passive loss of constitutive proteins upon *Gata3* deletion. Specifically, *Gata3* sufficiency under HFD does not simply preserve a baseline adipocyte proteome, it actively drives a gain-of-function inflammatory program. Consequently, the aGata3KO phenotype is better characterized by the absence of this acquired inflammatory signature than by generalized erosion of adipocyte protein content.

Consistent with this model, proteins elevated in control eWAT were strongly enriched for inflammatory and immune-related functions, pointing to an active, myeloid-driven inflammatory program sustained by *Gata3* under obesogenic conditions. Canonical markers of activated myeloid cells and tissue remodeling—including Itgax (CD11c), Lgals3, Ly86, Mmp12, Ctsk, and Atp6v0d2—were significantly suppressed in knockout mice. These proteins collectively span macrophage activation (Itgax, Lgals3, Ly86), lysosomal-mediated matrix degradation (Ctsk, Atp6v0d2), and extracellular matrix remodeling (Mmp12), delineating a coordinated inflammatory effector module rather than isolated hits. Reinforcing this interpretation, Hk3, a glycolytic enzyme associated with metabolic reprogramming during inflammatory macrophage polarization [34], was markedly decreased in knockout tissue, suggesting attenuation of the pro-inflammatory glycolytic switch that sustains myeloid cell function in inflamed depots. Together, these findings indicate that *Gata3* loss substantially dismantles the obesity-associated inflammatory network within eWAT, reducing both effector machinery and the metabolic infrastructure that supports it.

Against this backdrop of suppressed inflammation, *Gata3*-deficient adipose tissue displayed reciprocal induction of proteins governing glucose utilization and intermediary metabolism, consistent with release of the metabolic suppression typically imposed by chronic inflammatory signaling. Increased abundance of Slc2a4 (GLUT4) indicates enhanced capacity for insulin-stimulated glucose uptake, while concurrent upregulation of downstream glycolytic enzymes and metabolic regulators—Pklr, Ldhb, Gpd2, and Pck1—suggests increased glycolytic flux coupled to greater substrate flexibility. The co-elevation of Ldhb and Gpd2 is particularly informative: Ldhb preferentially converts lactate to pyruvate, facilitating entry into the TCA cycle, while Gpd2 encodes the mitochondrial glycerol-3-phosphate dehydrogenase, a core component of the glycerol–phosphate shuttle that regenerates cytosolic NAD^+^ and transfers reducing equivalents to the electron transport chain [35]. Their coordinated induction therefore implies enhanced coupling of cytosolic carbohydrate catabolism to mitochondrial oxidative metabolism, a hallmark of metabolically active, oxidative adipose tissue rather than glycolytically stressed tissue.

This reciprocal remodeling extended to lipid metabolic pathways. In control mice, elevated expression of Plin2, a lipid droplet-coating protein whose abundance scales with droplet size and lipid load, together with Soat1, which catalyzes cholesterol esterification for intracellular storage, is consistent with a lipid-accumulating, hypertrophic adipocyte state. In contrast, knockout mice exhibited increased expression of Mogat1, Abhd3, and Acaca, involved in monoacylglycerol acylation, phospholipid remodeling, and de novo fatty acid synthesis, respectively [36]. Rather than indicating net lipid accumulation, this pattern is more consistent with active lipid turnover and membrane remodeling, distinct from the pathological lipid burden characteristic of obese adipose tissue [37].

To further resolve this metabolic phenotype, we examined proteins governing mitochondrial function and oxidative capacity. Multiple structural and functional components of electron transport chain Complex I—Ndufb3, Ndufb7, Ndufb11, and Ndufs4—were elevated in knockout mice, alongside Coq4 and Coq7, rate-limiting enzymes in coenzyme Q biosynthesis, the electron carrier linking Complexes I and II to Complex III [38,39]. This coordinated upregulation implies a concerted expansion of oxidative phosphorylation capacity rather than incidental protein changes. Supporting this interpretation, proteins mediating complementary mitochondrial functions were similarly elevated: mitochondrial ribosomal subunits (Mrps21, Mrps30), indicating increased capacity for in situ synthesis of ETC subunits encoded by the mitochondrial genome; fatty acid oxidation enzymes (Acad8, Ivd, Slc25a21), suggesting augmented import and catabolism of acyl substrates [40]; antioxidant and detoxification proteins (Gsta3, Suox, Cyp2e1), reflecting adaptive management of reactive oxygen species generated by heightened oxidative flux [41,42]; and Atg101, indicative of upregulated mitophagy and mitochondrial quality control [43]. Collectively, these changes describe a coherent program of mitochondrial biogenesis, expanded oxidative capacity, and adaptive homeostasis.

These proteomic findings were reinforced by orthogonal system-level analyses. Pathway enrichment across GO Biological Process, Reactome, and KEGG databases independently identified innate immune activation, neutrophil-associated processes, and cell surface interactions at the vascular wall as dominant features of the control-enriched proteome (Figure 5F). Protein–protein interaction network analysis further resolved these signals into discrete modular architecture, including a densely interconnected inflammatory/macrophage/neutrophil module (Figure 5G, Supplement Figure S1). Within this module, hub analysis identified a small set of highly connected proteins whose loss in knockout tissue is predicted to propagate broadly within the network (Figure 5H). Transcription factor enrichment nominated STAT3 as the dominant upstream regulator of the control-enriched inflammatory program, with secondary contributions from IRF1, PU.1, and NFYA, factors linked to interferon-responsive transcription, myeloid lineage specification, and structural gene regulation, respectively (Figure 5I,J).

By contrast, the KO-enriched proteome defined a coherent metabolic reorientation rather than a passive mirror image. Both Enrichr and gProfiler concordantly identified mitochondrial Complex I biogenesis, electron transport chain activity, aerobic respiration, and branched-chain amino acid catabolism as dominant enriched categories (Figure 5K,L). Hub proteins within these clusters occupied positions of high centrality (Figure 5M), suggesting that metabolic remodeling downstream of *Gata3* loss is coordinated through a limited set of regulatory nodes. Together, the proteomic landscape defines a functional dichotomy: *Gata3* sufficiency drives a STAT3-dependent inflammatory state of high energetic cost, whereas its deletion permits a coordinated transition toward mitochondrial efficiency and enhanced oxidative metabolism, uncoupling adiposity from the cellular dysfunction that underlies systemic insulin resistance. This *Gata3*-dependent shift from an energetically costly inflammatory state toward enhanced mitochondrial efficiency and oxidative metabolism is mirrored at the level of metabolites and immune cell composition, as revealed by our subsequent metabolomic and mass cytometric analyses.

### 3.5. Adipocyte Gata3 Deficiency Coordinately Remodels the Adipose Proteome and Metabolome During Obesity

The proteomic signature of enhanced oxidative capacity raised the question of whether *Gata3* loss also produces a broader shift in small-molecule metabolism. To assess this, we performed targeted metabolomic profiling of visceral adipose tissue from HFD-fed control and aGata3KO mice. A targeted LC–MS/MS platform quantified 219 metabolites spanning central carbon, amino acid, nucleotide, redox, and lipid metabolism. The unsupervised analysis revealed reproducible, genotype-dependent differences, indicating coordinated metabolic remodeling rather than stochastic variation.

**Figure 6:**
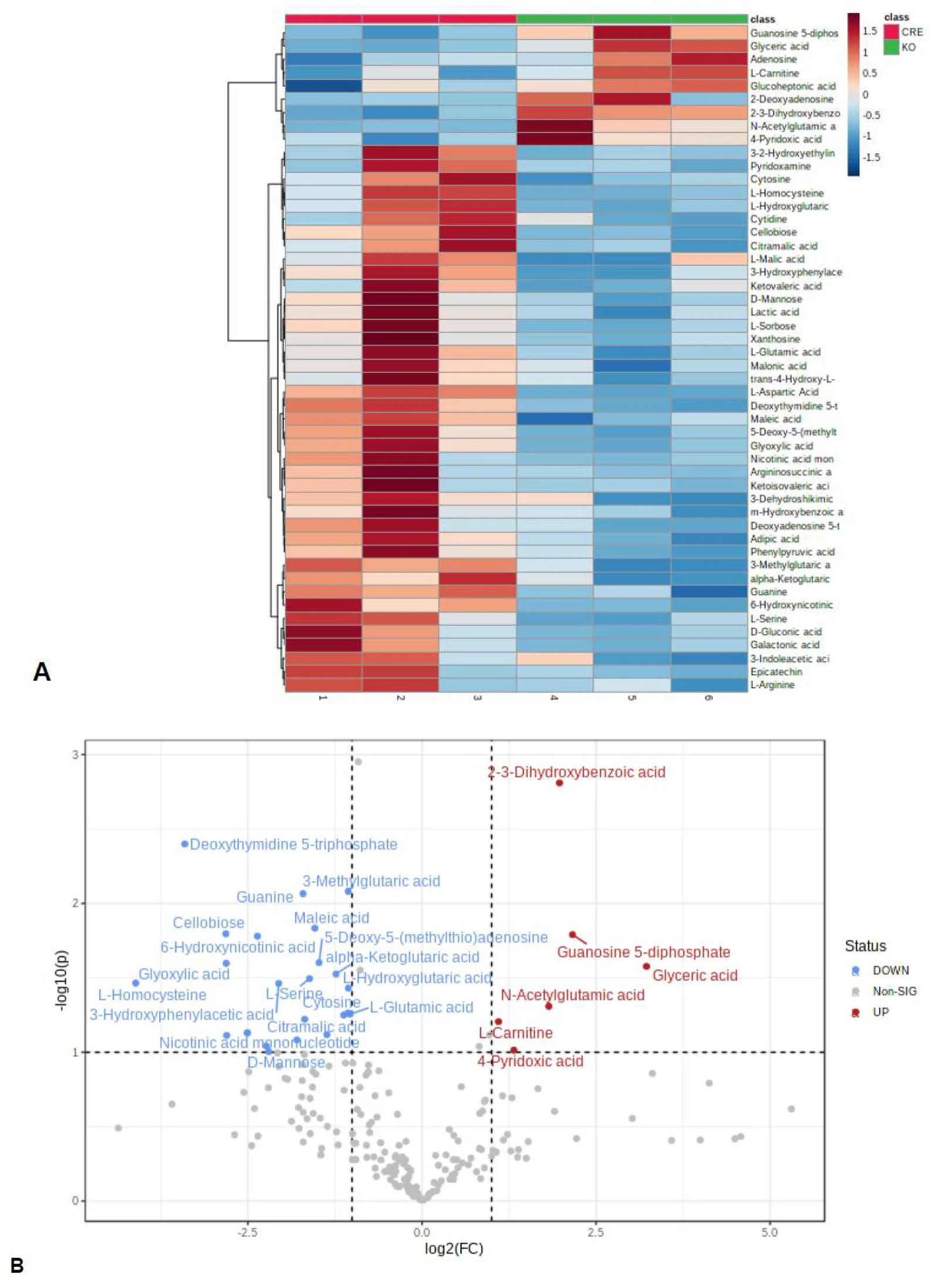

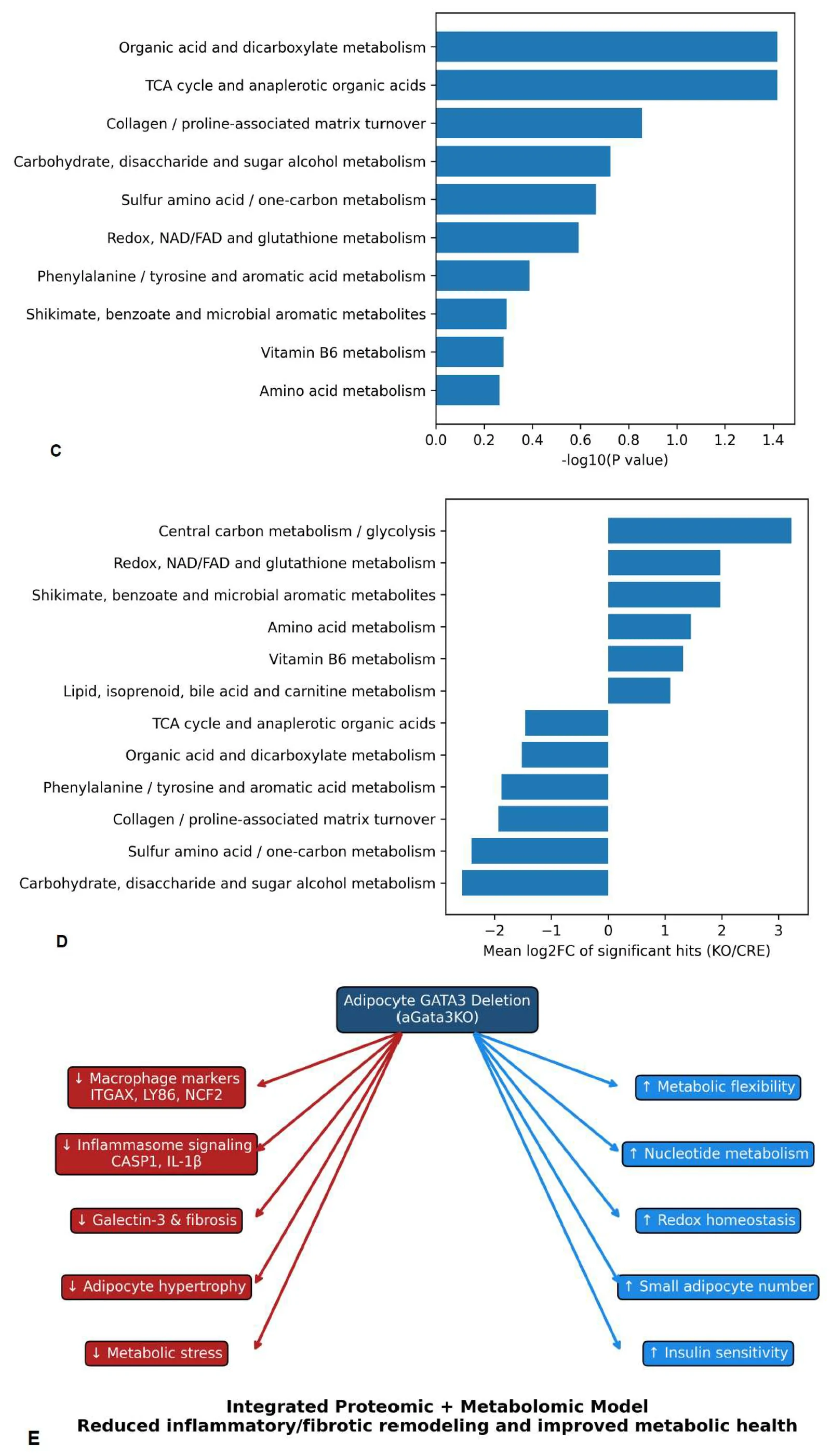
Adipocyte Gata3 deficiency promotes a metabolically adaptive adipose state. (**A**), Heat map of differentially abundant metabolites in visceral adipose tissue from control and aGata3KO mice. Metabolite abundances were normalized and row-standardized (Z score) before unsupervised hierarchical clustering. Rows represent metabolites and columns represent biological samples (control, n = 3; aGata3KO, n = 3). Warmer colors indicate higher relative abundance and cooler colors indicate lower relative abundance. Metabolites are annotated by biochemical class, highlighting coordinated, genotype-dependent remodeling of central carbon metabolism. (**B**), Volcano plot of differential metabolite abundance between control and aGata3KO mice. The x-axis shows log_2_ fold change (aGata3KO/control) and the y-axis shows −log_10_ (*p* value). Significantly increased metabolites are shown in red, significantly decreased metabolites in blue, and non-significant metabolites in gray. (**C**), Top enriched metabolite sets identified by mapping significantly altered metabolites to curated metabolic pathways. (**D**), Directionality of enriched metabolite sets, shown as the mean log_2_ fold change of significant metabolites within each pathway; positive values indicate enrichment in aGata3KO mice and negative values indicate depletion. (**E**), Integrated proteomic–metabolomic model summarizing reduced inflammatory/fibrotic remodeling and improved metabolic health in aGata3KO mice. Statistical analysis: relative metabolite abundances were determined by LC–MS/MS; differential metabolites were identified by two-sided unpaired Student’s *t*-test; metabolite set enrichment analysis was performed using over-representation analysis.

Unsupervised principal component analysis showed clear segregation of control and aGata3KO samples, with the first two principal components explaining 90.2% of total variance (PC1 = 69.6%; PC2 = 20.6%), indicating extensive metabolic reprogramming in *Gata3*-deficient adipose tissue. Hierarchical clustering similarly separated knockout and control samples into distinct clusters, demonstrating high within-group reproducibility and consistent genotype-dependent metabolic signatures (Figure 6A).

Volcano plot analysis demonstrated clear separation of metabolites as a function of genotype. The most significantly elevated metabolite in aGata3KO tissue was 2,3-dihydroxybenzoic acid (3.9-fold increase, *p* = 0.0016), whereas deoxythymidine 5′-triphosphate (dTTP), cellobiose, glyoxylic acid, L-homocysteine, guanine, and several additional metabolites were markedly reduced (Figure 6B). Fold-change analysis further revealed coordinated increases in adenosine 5′-diphosphate, inosine 5′-diphosphate, orotic acid, glyceric acid, N-acetylglutamic acid, and guanosine 5′-diphosphate, accompanied by reductions in D-maltose, glyoxylic acid, L-sorbose, nicotinic acid, L-homocysteine, and multiple deoxyribonucleotide intermediates. Rather than isolated biochemical changes, these patterns define several coherent metabolic programs. Elevated adenosine, inosine, and guanosine diphosphates together with orotic acid and N-acetylglutamic acid suggest enhanced nucleotide metabolism and remodeling of pyrimidine biosynthesis and nitrogen handling [44,45], while reductions in dTTP, guanine, cytosine, deoxyadenosine triphosphate, and related deoxyribonucleotides point to altered nucleotide turnover and DNA precursor metabolism [46]. Concurrent changes in glyceric acid, α-ketoglutarate, glyoxylic acid, citric acid, and gluconate implicate rewiring of central carbon metabolism, TCA cycle activity, and redox homeostasis [47]. Notably, reduced L-homocysteine is consistent with attenuation of one-carbon metabolism and oxidative stress [48], whereas increased L-carnitine suggests enhanced mitochondrial fatty acid utilization [49].

Metabolite set enrichment analysis (MSEA) supported these pathway-level inferences. Leading signals converged on carbohydrate and sugar alcohol metabolism, deoxyribonucleotide turnover, pyrimidine metabolism, organic acid metabolism, and redox/NAD-associated pathways (Figure 6C). Directional analysis showed enrichment of nucleotide and nitrogen-handling intermediates among KO-increased metabolites, whereas KO-decreased metabolites were enriched for deoxyribonucleotide, sugar, and organic acid features (Figure 6D). Together, these results support a model in which adipocyte *Gata3* deficiency shifts adipose metabolism away from obesogenic metabolic stress and toward a more adaptive state characterized by improved substrate handling and reduced redox burden.

Integration of the metabolomic and proteomic datasets reveals a remarkably coherent biological response to adipocyte *Gata3* deletion (Figure 6E). Suppression of inflammatory macrophage and matrix-remodeling proteins coincided with depletion of metabolites associated with glycolytic overflow, oxidative stress, and one-carbon metabolism, while enrichment of proteins involved in glucose transport, mitochondrial respiration, and lipid turnover parallel increased abundance of nucleotide and nitrogen-cycle intermediates. Collectively, these findings indicate that *Gata3* deficiency redirects adipose tissue away from chronic inflammatory and fibrotic remodeling toward a metabolically flexible state characterized by improved mitochondrial substrate utilization, enhanced nucleotide homeostasis, and reduced metabolic stress. These molecular signatures are concordant with the physiological phenotype of aGata3KO mice, reduced adipocyte hypertrophy, diminished adipose inflammation and fibrosis, improved insulin sensitivity, and resistance to diet-induced obesity, and together identify *Gata3* as a central regulator of obesity-associated adipose dysfunction whose deletion coordinately reprograms both the adipose proteome and metabolome toward a healthier state.

### 3.6. Single-Cell Mass Cytometry Reveals Gata3-Dependent Multi-Lineage Immune Infiltration in Obese Adipose Tissue

The proteomic identification of STAT3-driven myeloid signatures generated a direct, testable prediction: if *Gata3* sufficiency actively sustains this program, then its deletion should produce measurable, multi-lineage contraction and reprogramming of the adipose immune landscape. To examine this, we performed mass cytometry by time of flight (CyTOF), which allows simultaneous measurement of multiple parameters at the single-cell level [50], on CD45^+^ cells from the stromal vascular fraction (SVF) of inguinal (iWAT) and gonadal (eWAT) adipose tissue from HFD-fed Adipoq-Cre and aGata3KO mice, pooling cells from five mice per group to enable high-dimensional, depot-integrated phenotyping.

**Figure 7:**
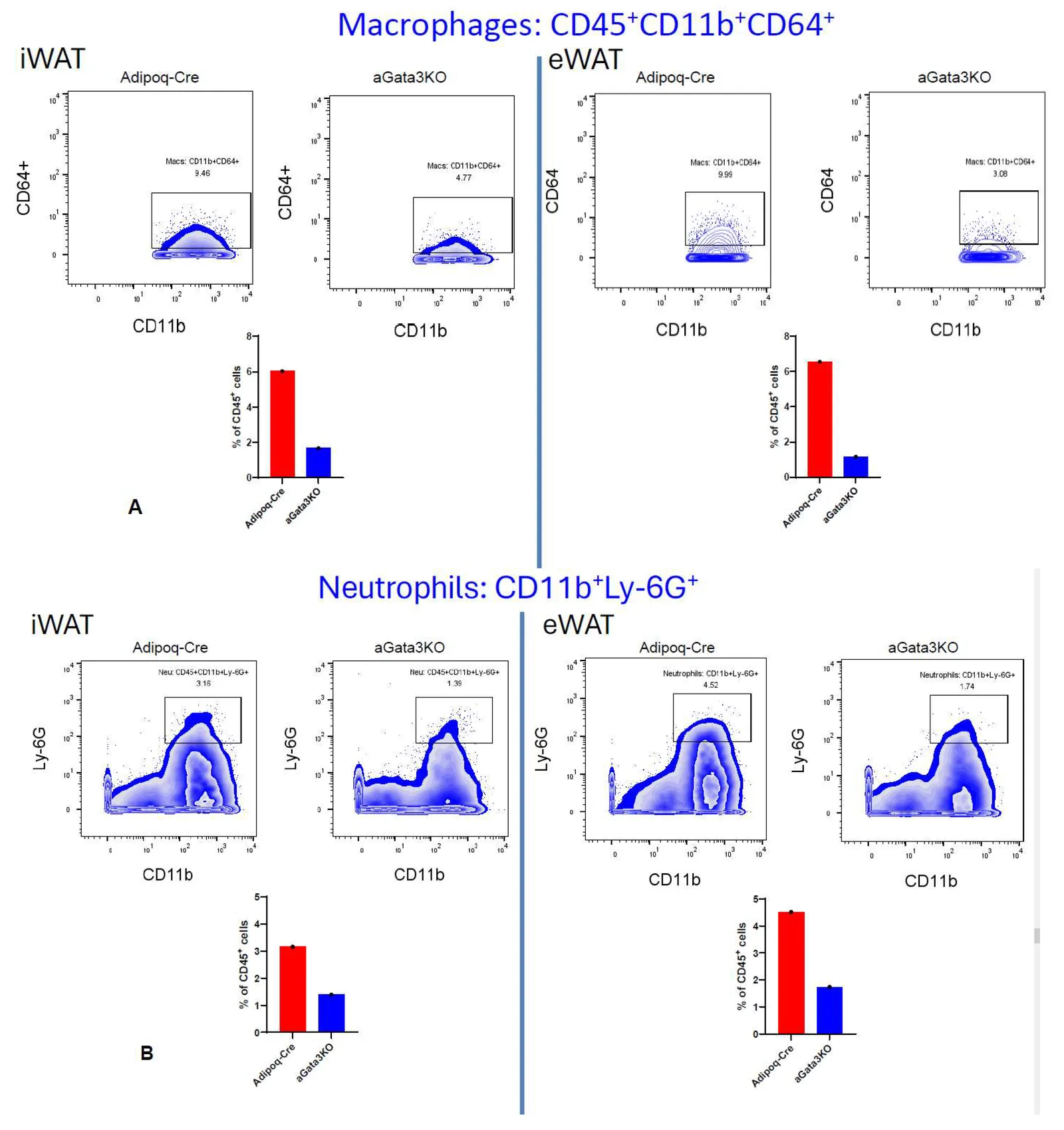

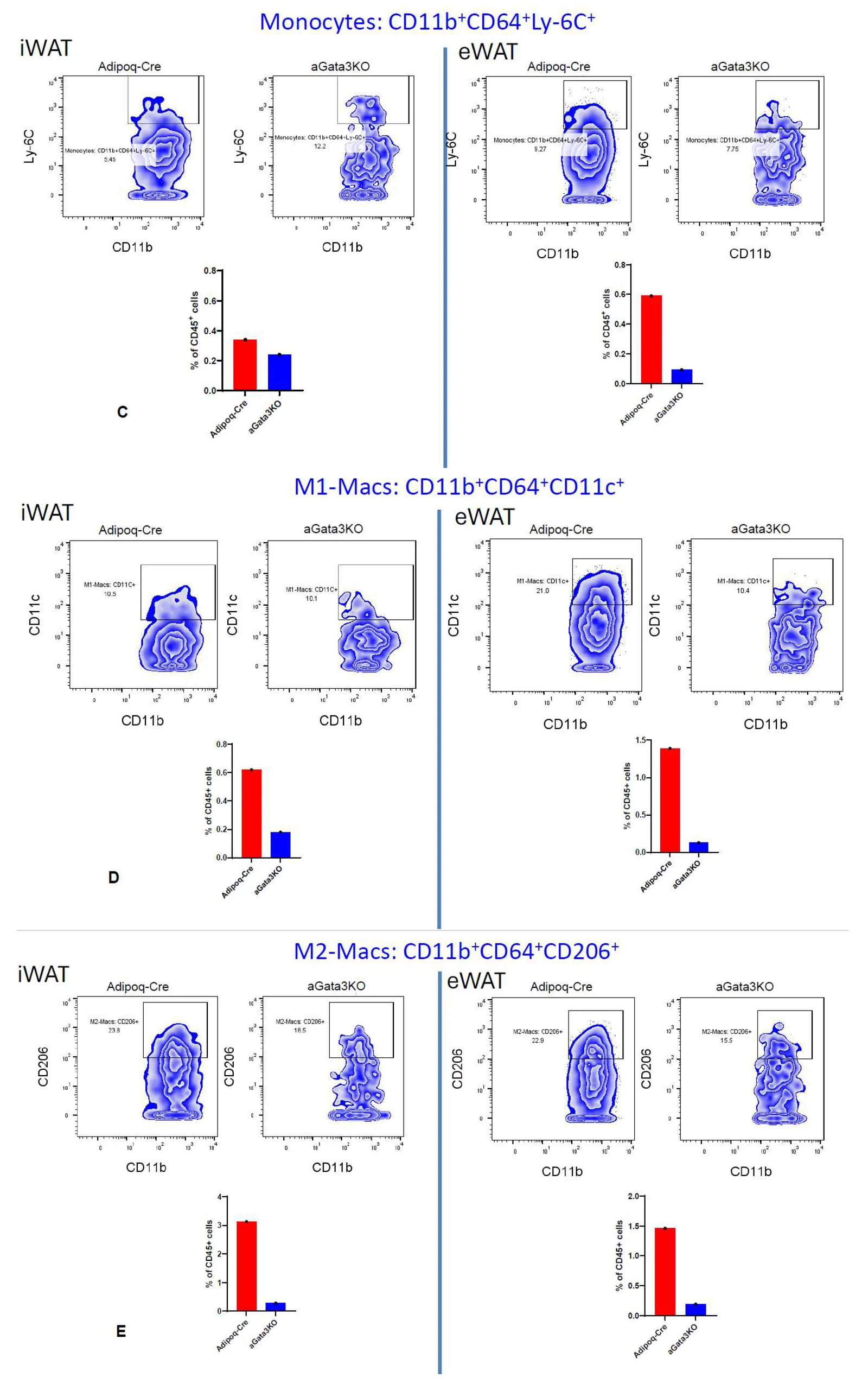

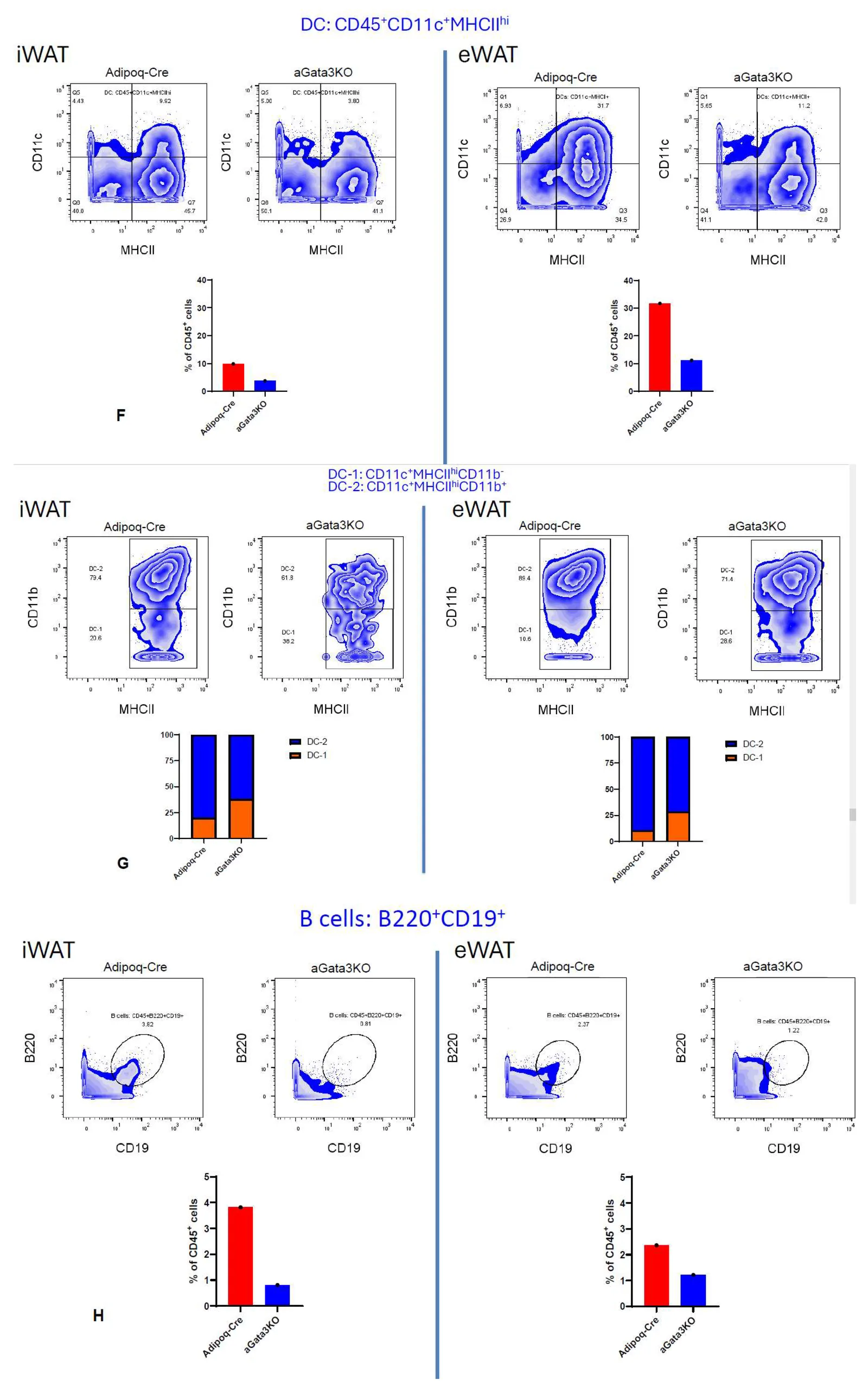

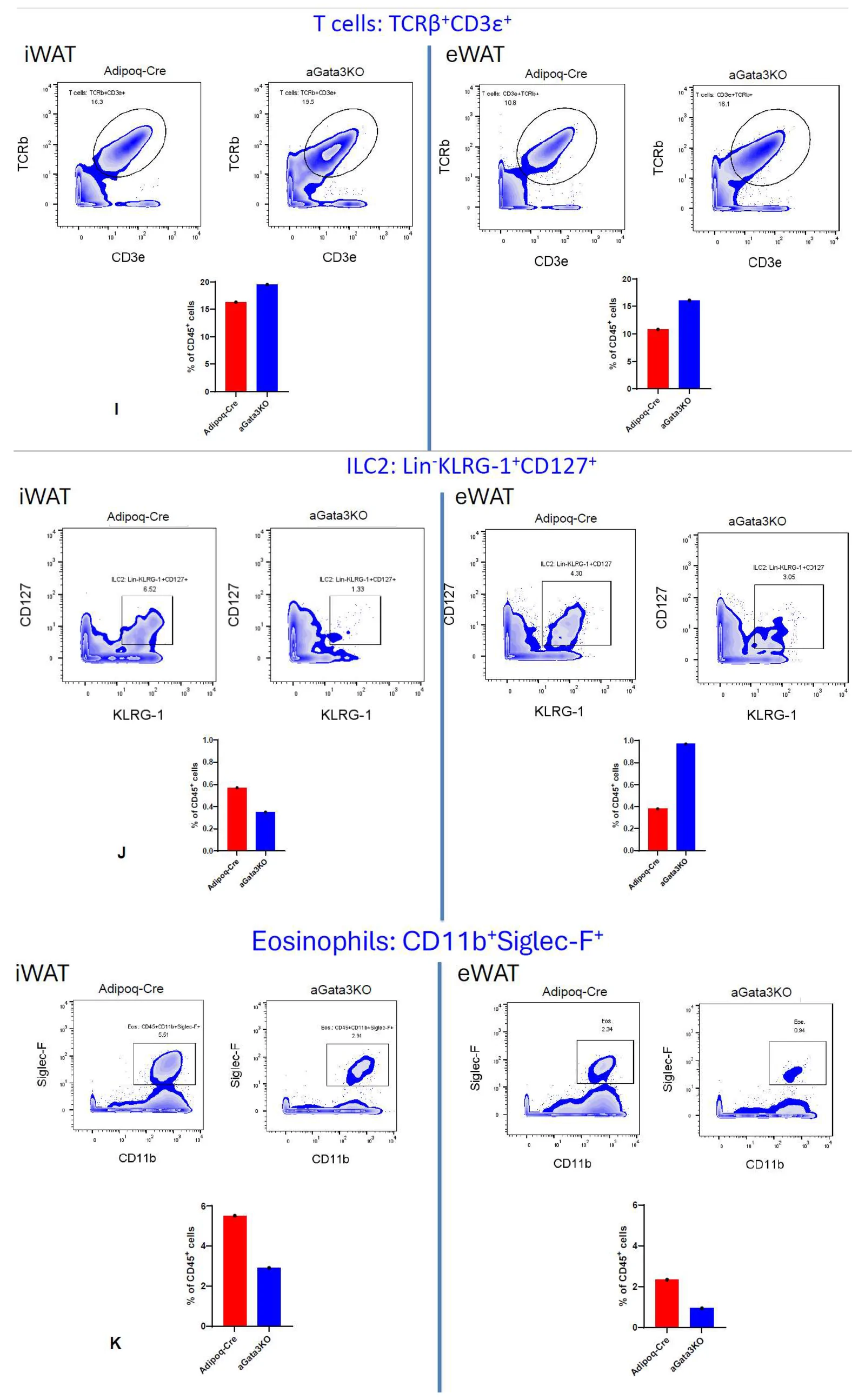
Mass cytometry reveals broad immune remodeling following adipocyte Gata3 deletion. Inguinal (iWAT) and epididymal (eWAT) adipose depots were isolated from Adipoq-Cre and aGata3KO mice after 16 weeks of HFD. Depots were digested, SVF cells were prepared, and cells from each genotype were pooled (n = 3 mice per genotype) for CyTOF analysis with the indicated antibody panel. (**A**), Macrophages (CD45^+^CD11b^+^CD64^+^). (**B**), Neutrophils (CD11b^+^Ly6G^+^). (**C**), Monocytes (CD11b^+^CD64^+^Ly6C^+^). (**D**,**E**), M1-like and M2-like macrophage subsets. (**F**,**G**), Dendritic-cell populations, including CD11b^−^ and CD11b^+^ subsets. (**H**), B cells (B220^+^CD19^+^). (**I**), T cells (TCRβ^+^CD3ε^+^). (**J**), Group 2 innate lymphoid cells (ILC2s). (**K**), Eosinophils (CD11b^+^Siglec-F^+^). Quantifications of CyTOF data were plotted for each fat depot and cell type.

CyTOF profiling revealed profound, depot-consistent immune remodeling in aGata3KO mice, in line with the proteomic prediction. Total CD45^+^ frequency was reduced by ~45% relative to controls (fold change = −0.75; *p* < 0.01), a magnitude incompatible with isolated perturbation of a single immune population and instead indicative of coordinated dismantling of the broader inflammatory architecture. This global contraction was driven predominantly by reductions in three pro-inflammatory myeloid populations: CD11b^+^/CD64^+^ macrophages (Figure 7A), CD11b^+^/Ly6G^+^ neutrophils (Figure 7B), and CD11b^+^/CD64^+^/Ly6C^+^ monocytes (Figure 7C), the primary cellular effectors of chronic adipose inflammation and paracrine insulin resistance [51]. Their coordinated reduction across distinct lineages establishes that *Gata3* sustains a redundant, multi-cellular innate inflammatory program rather than acting through dependence on any single effector population. Flow-cytometric analysis of individual mice further corroborated the macrophage findings (Supplement Figure S2).

Beyond quantitative contraction, *Gata3* deficiency also produced qualitative shifts in myeloid identity, indicating that the residual immune compartment is not merely smaller but functionally reoriented. M1-like (CD11c^+^) and M2-like (CD206^+^) macrophage subsets were reduced by 55% and 35%, respectively (FDR < 0.05; Figure 7D,E), with the disproportionately greater loss of M1-like cells skewing the residual macrophage pool toward a less inflammatory phenotype independent of total macrophage abundance. This reorientation extended to dendritic cells: pro-inflammatory CD11b^+^ DCs fell by 60% while anti-inflammatory CD11b^−^ DCs increased by ~20% (Figure 7F,G), reversing the obesity-associated displacement of homeostatic resident DCs by recruited pro-inflammatory subsets. Taken together, these parallel changes across macrophage and DC lineages indicate that *Gata3* loss not only reduces myeloid cell number but actively rebalances myeloid identity toward a homeostatic state.

The lymphoid compartment was similarly, though more heterogeneously, remodeled. B220^+^/CD19^+^ B cells were reduced by 50% in aGata3KO mice (FDR < 0.01; Figure 7H), attenuating a B cell-driven amplification loop that sustains macrophage activation via IgG-mediated signaling and pro-inflammatory cytokine production [52]. TCRβ^+^/CD3ε^+^ T cells showed a modest numerical increase, but their surface phenotype was consistent with functional quiescence rather than inflammatory activation (Figure 7I), suggesting that this expansion occurs in a non-pathogenic direction in the context of reduced myeloid inflammatory drive [53]. ILC2 frequency was reduced in iWAT but increased in eWAT of aGata3KO mice (Figure 7J), a depot-divergent pattern likely reflecting intrinsic differences in baseline inflammatory tone between subcutaneous and visceral depots rather than a uniform systemic effect of *Gata3* loss. Eosinophils (SiglecF^+^) were reduced across both depots (Figure 7K); although eosinophils are classically considered anti-inflammatory in adipose tissue, this reduction is more plausibly explained by diminished upstream ILC2-driven recruitment, proportional to the overall contraction of type 2 and myeloid responses, than by a primary defect in eosinophil function [54].

Together, the CyTOF data demonstrate that *Gata3* deficiency induces comprehensive, multi-lineage restructuring of the adipose immune landscape: contracting the myeloid axis, reorienting residual myeloid cells toward less inflammatory phenotypes, attenuating B cell-driven amplification, and rescaling the type 2 lymphoid response in proportion to reduced inflammatory burden. This cellular remodeling aligns with the proteomic evidence for suppressed STAT3 signaling and loss of macrophage/neutrophil identity proteins, providing cross-platform validation of *Gata3* as an active requirement for the inflammatory microenvironment that drives adipose dysfunction in obesity.

### 3.7. Adipocyte Gata3 Promotes Hepatic Dysfunction Through Coordinated Remodeling of the Circulating Lipidome and Chemokine Milieu

**Figure 8:**
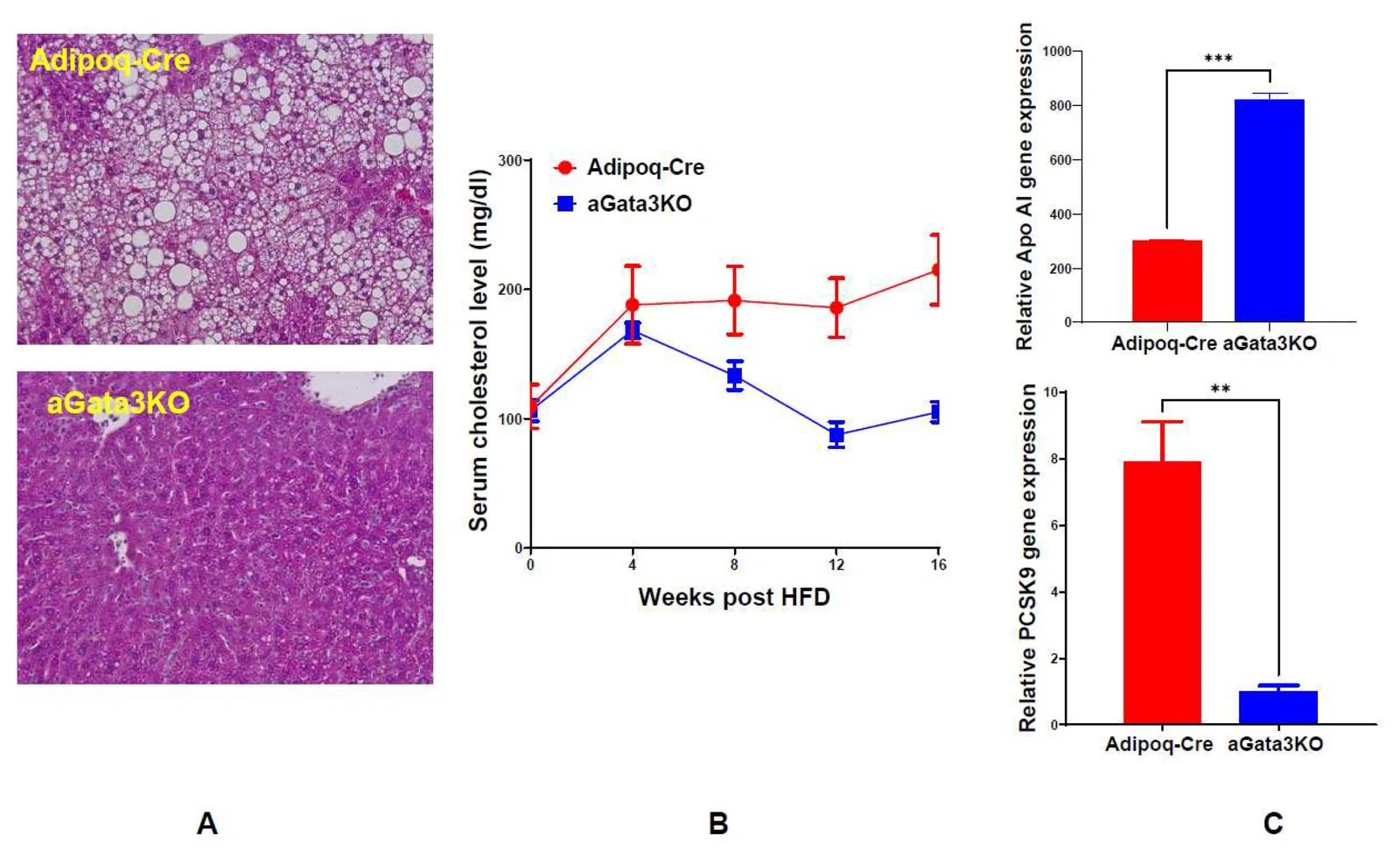

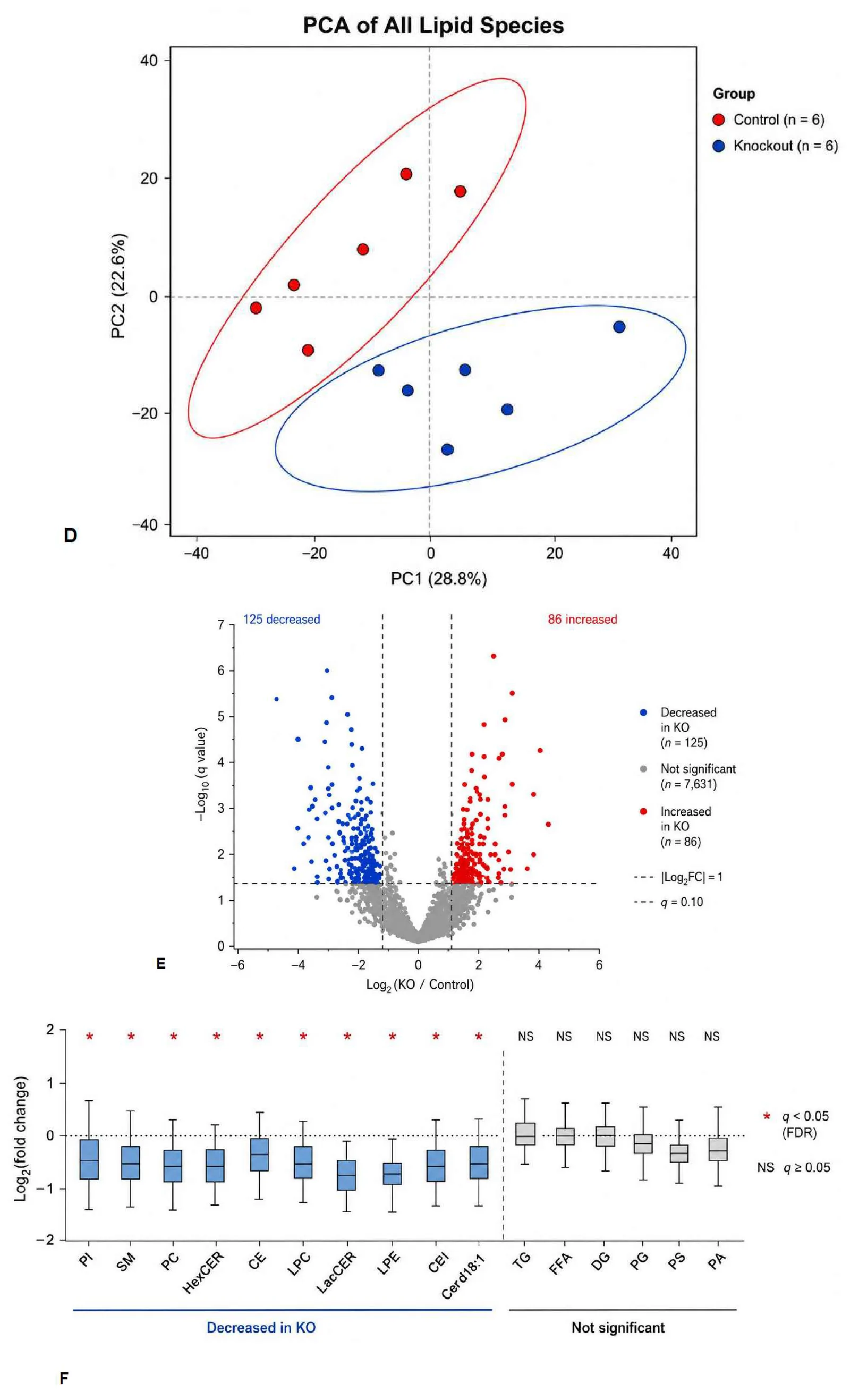

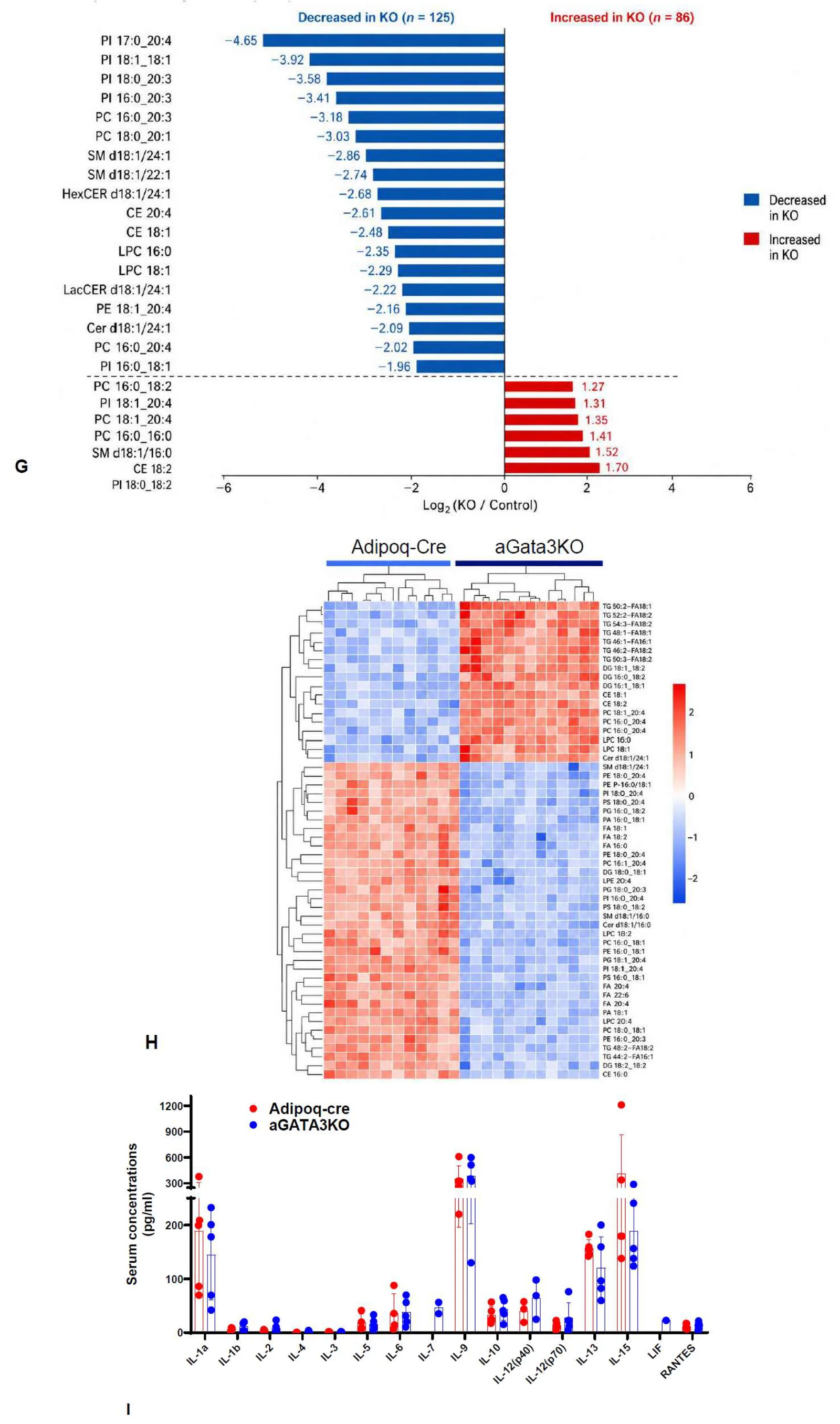

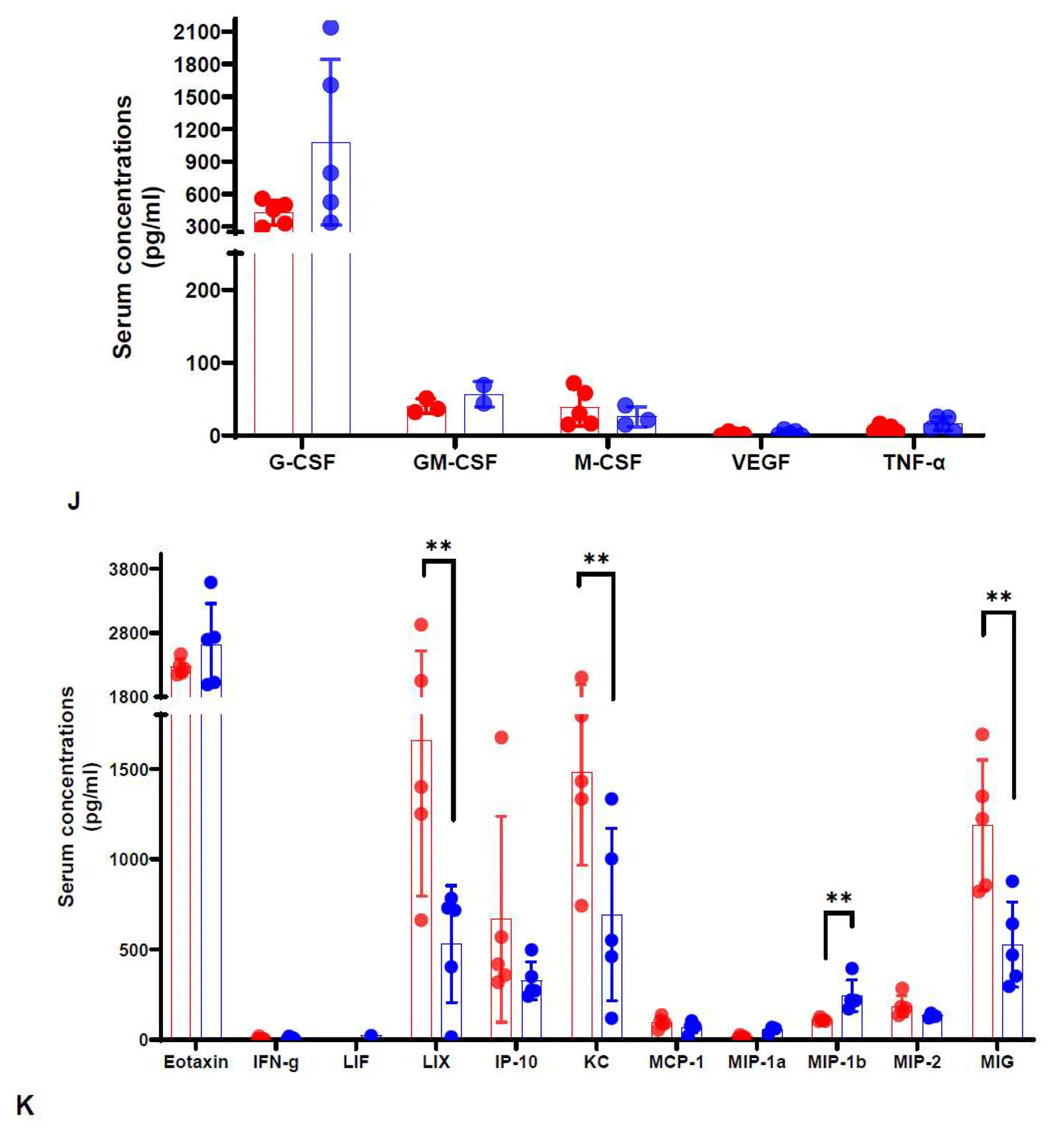
GATA3 deficiency attenuates hepatic steatosis and remodels the circulating lipidome and chemokine pathways. (**A**), Representative H&E-stained liver sections from control and aGata3KO mice after HFD feeding, showing reduced hepatic steatosis in aGata3KO mice. (**B**), Serum cholesterol concentrations in control and aGata3KO mice during HFD feeding, measured by ELISA (n = 16 mice per genotype). (**C**), Hepatic *Apoa1* and *Pcsk9* mRNA expression after 16 weeks of HFD, quantified by qPCR from total liver RNA using gene-specific primers. (**D**), Principal component analysis (PCA) of the combined plasma lipidomic datasets from Experiments 2 and 7, performed on normalized abundances of all quantified lipid species. Each point represents one biological replicate (controls, light blue; knockouts, dark blue); ellipses denote 95% confidence intervals. (**E**), Volcano plot of all quantified plasma lipid species from the batch-adjusted linear model. The x-axis shows batch-adjusted log_2_ fold change (KO/control) and the y-axis shows −log_10_ (*p* value). Red points indicate lipid species significantly increased in knockout plasma (FDR q < 0.10), blue points indicate species significantly decreased (FDR q < 0.10), and gray points indicate non-significant species. Vertical dashed lines mark |log_2_ fold change| = 1; the horizontal dashed line marks *p* = 0.05. In total, 211 lipid species were significantly altered (125 decreased, 86 increased). (**F**), Box-and-whisker plots showing the distribution of log_2_ fold changes (KO/control) for all molecular species within each lipid class. Boxes denote median and interquartile range; whiskers represent 1.5 × IQR. Lipid classes significantly altered after batch adjustment and Benjamini–Hochberg correction (FDR q < 0.05) are shown in blue; non-significant classes are shown in gray. Positive values indicate enrichment in knockout mice; negative values indicate depletion. (**G**), Horizontal bar plot of the 24 lipid species with the largest significant genotype-dependent changes. Bars represent batch-adjusted log_2_ fold change (KO/control), with negative values indicating depletion and positive values indicating enrichment in knockout plasma; species are ranked by effect size. (**H**), Hierarchical clustering of the 50 most differentially abundant lipid species. The heat map shows row-wise Z-score-normalized abundances; columns correspond to individual samples and rows to lipid species. Unsupervised clustering (Euclidean distance, complete linkage) was applied to both samples and lipids. Red indicates relatively increased abundance and blue indicates relatively decreased abundance, with samples clustering predominantly by genotype. (**I**–**K**), Serum cytokine, hematopoietic growth factor, and chemokine concentrations in control and aGata3KO mice after 16 weeks of HFD, measured by Luminex xMAP^®^ magnetic bead immunoassay (n = 5 mice per genotype). Bars represent mean ± s.e.m.; each data point represents an individual mouse.

Dysfunctional adipose tissue is known to drive insulin resistance and hepatic steatosis through inflammatory spillover and the release of lipids, adipokines, and other circulating mediators that prime the liver toward steatotic, pro-inflammatory, and pro-fibrotic states [55]. We hypothesized that the adipocyte-intrinsic remodeling driven by *Gata3* loss would propagate systemically to influence the hepatic phenotype. Consistent with this hypothesis, aGata3KO mice showed hepatoprotection across multiple independent indices: reduced liver weight (Figure 3C), attenuated steatosis with preserved architecture (Figure 8A), lower serum cholesterol (Figure 8B), increased hepatic ApoA1, and decreased PCSK9 expression (Figure 8C). Because these indices span distinct pathophysiological domains, organ mass, histology, circulating lipids, and hepatic gene expression, their convergence indicates a coordinated hepatoprotective phenotype rather than an isolated or incidental effect, and motivates a systematic dissection of the systemic mediators responsible. The lipidomic and chemokine analyses below delineate the specific pathways through which adipocyte *Gata3* activity promotes or mitigates this hepatic dysfunction.

To determine how *Gata3* deficiency alters the circulating lipid milieu, we next analyzed the serum lipidome, pooling plasma lipidomic datasets from two independent cohorts (Experiments 2 and 7) to increase statistical power and assess reproducibility across biological replicates. Lipid abundance was log_2_-transformed, normalized, and modeled as a function of genotype using a batch-adjusted linear model that included the experimental cohort as a covariate, allowing genotype effects to be isolated from inter-experiment variation. Following batch adjustment, principal component analysis revealed clear genotype-dependent segregation, with PC1 and PC2 explaining 28.8% and 22.6% of the total variance, respectively (Figure 8D). Modest cohort-related differences remained detectable, but cohort correction rendered genotype, not cohort, the dominant axis of clustering, indicating that the lipidomic phenotype is reproducible across independent experiments rather than driven by batch-specific artifacts. Consistent with this genotype-driven segregation, volcano plot analysis revealed extensive remodeling of the plasma lipidome (Figure 8E): after Benjamini–Hochberg correction, 211 lipid species were differentially abundant between control and knockout mice (FDR q < 0.10). These significant species were broadly distributed across the volcano plot rather than concentrated among a small number of outliers, indicating that genotype-dependent changes span multiple lipid subclasses and reflect a global shift in lipid metabolic programming downstream of *Gata3*, rather than a localized perturbation of a single pathway.

At the lipid class level, nine classes remained significantly altered following batch adjustment and multiple-testing correction, all reduced in knockout plasma: phosphatidylinositol (PI), phosphatidylcholine (PC), sphingomyelin (SM), cholesterol esters (CE), hexosylceramides (HexCER), lysophosphatidylcholine (LPC), lactosylceramides (LacCER), lysophosphatidylethanolamine (LPE), and ceramides. In contrast, total triglycerides and free fatty acids, the principal neutral lipid pools, were unchanged (Figure 8F). This pattern indicates that the dominant alterations are confined to membrane phospholipids, sphingolipids, and lysophospholipid catabolic products, while bulk energy-storage lipids remain intact. This selectivity is mechanistically informative: rather than causing generalized dyslipidemia, *Gata3* deficiency appears to disrupt membrane and lipoprotein-surface lipid homeostasis specifically, implicating hepatic phospholipid biosynthesis or export, lipoprotein remodeling, and sphingolipid turnover as candidate downstream mechanisms. Two independent analyses reinforced this conclusion. First, effect-size ranking identified phospholipid, sphingolipid, cholesterol ester, and glycosphingolipid species as the most strongly altered features of the knockout lipidome (Figure 8G), consistent with the class-level pattern above. Second, hierarchical clustering of the fifty most variable species confirmed genotype-dependent segregation across individual samples from both cohorts (Figure 8H), demonstrating that this selectivity is not an artifact of averaging but holds at the level of individual animals. Because cholesterol esters and glycosphingolipids co-localize at lipoprotein particle surfaces and within membrane microdomains, where they govern particle stability and membrane curvature [56], their selective reduction points to coordinated remodeling of lipoprotein surface composition as a proximal consequence of *Gata3* loss. This defines a “lipid arm” of the mechanism, in which *Gata3*-deficient animals circulate a less lipotoxic lipoprotein particle that delivers fewer pro-inflammatory and pro-fibrotic signals to the liver.

In parallel with this lipid remodeling, we examined how adipocyte *Gata3* deficiency alters the circulating inflammatory milieu. Multiplex profiling of more than 30 serum immune mediators revealed a strikingly selective pattern: sixteen interleukins, including IL-1α, IL-1β, IL-6, IL-10, and IL-13, and five myeloid growth factors showed no significant genotype differences (Figure 8I,J). This absence of broad cytokine change indicates that *Gata3* deletion does not induce generalized immunosuppression, and instead points to a more restricted signaling axis; consistent with this, the dominant genotype-dependent effects were confined to chemokine signaling. Of eleven chemokines quantified, four reached significance (*p* < 0.01) and segregated into two opposing programs that mirror the CyTOF-defined immune remodeling (Figure 8K). Three were elevated in control serum and formed a coordinated neutrophil–Th1 axis: CXCL5 was ~3-fold higher in controls, implicating *Gata3*-dependent CXCL5 as a systemic bridge linking adipocyte dysfunction to neutrophil recruitment; CXCL2 was co-elevated, providing a redundant ELR-positive neutrophil-homing signal; and CXCL9 was ~2.3-fold higher, consistent with active IFN-γ signaling and sustained Th1 activation [57]. Conversely, CCL3 and CCL4 were elevated in aGata3KO serum, reflecting a shift away from this self-amplifying neutrophil–Th1 program and toward monocyte- and lymphocyte-directed homeostatic surveillance [58], a pattern concordant with the reduced myeloid infiltration and loss of macrophage identity proteins observed by CyTOF and proteomics, respectively.

Integrated with the lipidomic data above, these findings define a two-armed mechanism by which adipocyte *Gata3* promotes hepatic dysfunction. In the lipid arm, *Gata3*-dependent adipocyte hypertrophy generates a pro-lipotoxic lipoprotein surface enriched in cholesterol esters and glycosphingolipids. In the chemokine arm, *Gata3*-driven CXCL5/CXCL2/CXCL9 signaling recruits neutrophils and Th1 cells that prime hepatic cells toward pro-inflammatory, pro-fibrotic states. Because adipocyte *Gata3* deletion dismantles both arms simultaneously, it attenuates hepatic inflammatory tone through two convergent routes rather than one, positioning *Gata3* as a central coordinator of systemic lipid and chemokine spillover that links adipocyte-intrinsic dysfunction to multi-organ metabolic disease.

## 4. Discussion

The prevailing model of adipose transcriptional regulation has long positioned *Gata3* as a canonical repressor of adipogenesis, a brake on the *Pparγ*-centered hierarchy, such that its loss was predicted to enhance adipocyte formation and exacerbate metabolic outcomes. Our findings revise this framework comprehensively. Using adipocyte-restricted genetic tools with cell-autonomous precision unavailable to prior approaches, we show that *Gata3* is dispensable for basal adipogenesis but becomes an essential driver of pathological adipose expansion and systemic metabolic dysfunction under chronic caloric excess. *Gata3* is therefore not a constitutive anti-adipogenic brake but a diet-inducible regulator whose activity in mature adipocytes is selectively activated to coordinate maladaptive remodeling in obesity.

This reinterpretation is warranted because the anti-adipogenic paradigm rests on three methodologically limited foundations: preadipocyte cell lines that incompletely recapitulate in vivo adipocyte chromatin architecture and transcriptional state; promoter-occupancy data that establish binding but not transcriptional directionality, particularly for a factor now recognized as a pioneer transcription factor capable of activating or repressing targets depending on chromatin context; and non-specific in vivo perturbations that alter *Gata3* expression across all adipose cell types simultaneously. Because non-adipocytes substantially outnumber mature adipocytes within adipose tissue and *Gata3* is broadly expressed across stromal and immune compartments, these prior strategies could not isolate adipocyte-intrinsic function, raising the possibility that reported anti-adipogenic effects reflect paracrine signaling from non-adipocyte populations rather than direct *Gata3* action within the adipocyte itself.

The aGata3KO phenotype is structurally incompatible with a constitutive repressor model on its own logic: if *Gata3* simply suppressed *Pparγ* and adipogenesis, its deletion should increase adiposity and aggravate metabolic outcomes. Instead, adipocyte-specific *Gata3* loss protects against diet-induced obesity, redirecting adipose expansion from hypertrophic to hyperplastic growth and improving glucose tolerance and insulin sensitivity while reducing adipose inflammation and fibrosis. This phenotype is not merely inconsistent with a repressor model; it is the precise inverse of what that model predicts. Rather than a passive brake whose removal accelerates adipogenesis, *Gata3* emerges as an active promoter of the maladaptive hypertrophic program, one that must be present, and functionally engaged by nutritional excess, for pathological adipose expansion to proceed.

The depot-specific and nutritionally dynamic *Gata3* expression pattern reported here also reconciles our findings with a recent report in which systemic *Gata3* suppression via liposomal delivery produced no discernible changes in fat depot distribution or organ weights [14]. As detailed in the Results, apparent *Gata3* expression changes in whole-tissue mRNA preparations reflect a cell-composition confound—progressive dilution of the adipocyte-derived signal by an expanding immune-cell GAPDH denominator—rather than genuine transcriptional repression in adipocytes. This confound is most pronounced in the inflamed visceral depots sampled by the liposomal approach, and is absent in the inguinal subcutaneous depot where immune infiltration is lower and apparent expression correspondingly increases. Liposomal delivery is additionally constrained by its non-selective cellular reach, which precludes isolation of adipocyte-intrinsic function and obscures the depot-specific and nutritionally regulated pattern our genetic model resolves. The adipocyte-restricted precision of the aGata3KO and aGata3KI models therefore does not contradict that null result but reveals a layer of biological specificity that non-selective delivery approaches are structurally incapable of detecting.

The humanized knock-in experiment extends loss-of-function findings by establishing causal sufficiency and cross-species conservation simultaneously. Adipocyte-restricted re-expression of human GATA3 on the knockout background fully restored weight gain, adipose expansion, glucose intolerance, insulin resistance, and hyperleptinemia while preserving adiponectin expression, reinstating pathology without sacrificing adipocyte identity. This bidirectional result that loss-of-function protects and gain-of-function restores pathology satisfies both necessity and sufficiency criteria for a causal driver. That this reconstitution is achieved by the human ortholog rather than the murine sequence establishes that the pathogenic function of GATA3 in mature adipocytes is conserved across species, directly grounding the mechanistic findings in a human-relevant context.

Multi-omics profiling resolves the mechanistic architecture downstream of *Gata3* and converges on a single integrated conclusion: adipocyte *Gata3* governs not only the abundance but the inflammatory identity of the adipose immune niche. At the molecular level, proteomics identifies a dominant gain of an inflammatory myeloid program, macrophage activation, lysosomal remodeling, and glycolytic reprogramming, with STAT3 as the strongest predicted upstream driver and IRF1 and PU.1 as secondary contributors, while simultaneously revealing a reciprocal oxidative program in *Gata3*-deficient adipose tissue marked by increased GLUT4, glycerol–phosphate shuttle enzymes, electron transport chain subunits, and mitochondrial quality-control components. At the metabolite level, metabolomics confirms this oxidative shift, with *Gata3* loss associated with reduced oxidative stress markers and one-carbon metabolites alongside increased intermediates consistent with mitochondrial substrate utilization. At the cellular level, CyTOF provides the biological interpretation of these molecular signatures: *Gata3* deficiency reduces total CD45 cells in the stromal vascular fraction through coordinated decreases in macrophages, neutrophils, and monocytes, and qualitatively rebalances the residual myeloid pool by disproportionately depleting M1-like relative to M2-like macrophages and reciprocally remodeling dendritic cell subsets. The three platforms are therefore not parallel but hierarchically explanatory: proteomics defines the transcriptional program, metabolomics confirms its metabolic output, and CyTOF identifies the cells responsible for both.

Systemic lipidomic and chemokine profiling identifies two mechanistically independent routes by which this adipocyte-intrinsic program produces hepatic dysfunction. In the lipid arm, *Gata3*-dependent remodeling of the circulating lipidome is selective, reducing membrane phospholipids, sphingolipids, and cholesterol esters, which localize predominantly to the lipoprotein surface monolayer and govern receptor engagement, immune recognition, and hepatic uptake, while sparing triglycerides and free fatty acids that constitute bulk lipid cargo. This selectivity points toward altered lipoprotein surface chemistry as the operative mechanism rather than a generic increase in lipid burden. In the chemokine arm, elevated CXCL5, CXCL2, and CXCL9 define a coordinated neutrophil and Th1 recruitment axis that implicates an inflammatory route linking adipose immune activation to hepatic priming, while the preservation of interleukins and myeloid growth factors argues against generalized immunosuppression and in favor of a specific, mechanistically tractable channel of interorgan communication. Together, these two arms constitute a coherent circuit through which adipocyte-intrinsic *Gata3* activity is converted into systemic metabolic and hepatic pathology via parallel lipid and immune effector streams, each independently tractable as a therapeutic target.

In summary, these findings establish *Gata3* as a context-dependent, diet-inducible coordinator of pathological adipose remodeling rather than a constitutive gatekeeper of adipocyte differentiation. In mature adipocytes under chronic nutrient excess, *Gata3* is both necessary and sufficient to drive hypertrophic expansion, immune remodeling, lipid dyshomeostasis, and hepatic dysfunction. This reframing from canonical repressor to inducible pathogenic driver carries direct therapeutic implications: because *Gata3* is dispensable for basal adipocyte function yet required for the maladaptive response to caloric excess, adipocyte-targeted GATA3 inhibition offers a mechanistic rationale for uncoupling adipose expansion from metabolic deterioration. The identification of discrete downstream effector arms, the CXCL5/CXCL2/CXCL9 chemokine axis and the lipoprotein surface lipidome, further nominates pharmacologically tractable nodes for selective intervention without requiring upstream GATA3 inhibition itself.

Two limitations qualify the scope of these conclusions. First, all phenotyping and multi-omic analyses were performed in male C57BL/6J mice. Female C57BL/6J mice show intrinsic resistance to HFD-induced obesity and adipose inflammation relative to males (Supplementary Figure S3), a phenotype that itself reflects lower visceral immune infiltration under the dietary conditions used, and consistently with this, female aGata3KO mice showed no significant genotype-dependent weight differences under the same HFD protocol [59,60,61,62,63]. This null result in females is therefore interpretively coherent rather than merely a sex limitation: it suggests that *Gata3*-dependent maladaptive remodeling requires a permissive pro-inflammatory adipose environment whose development is hormonally regulated and less robustly induced in females under standard HFD conditions. Whether *Gata3* plays an analogous role in female mice under more aggressive dietary challenge or in post-menopausal states, where the hormonal protection against adipose inflammation is reduced, remains to be determined. Second, the multi-omic profiling captures a single cross-sectional time point after established obesity, and cannot establish whether *Gata3* activation precedes or follows the onset of adipose inflammation.

## Data Availability

The original contributions presented in this study are included in the article/Supplementary Materials. Further inquiries can be directed to the corresponding author.

## Supplementary Materials

The following supporting information can be downloaded at: https://www.mdpi.com/article/10.3390/cells15181644/s1, Figure S1: g:Profiler pathway-enrichment analysis of adipose-tissue proteomic changes in aGata3KO mice; Figure S2: Adipocyte-specific Gata3 deficiency reduces macrophage content in epididymal adipose tissue; Figure S3: Gata3 deficiency does not alter weight gain in female mice fed a high-fat diet; Table S1: List of qPCR primers; Table S2: List of Antibodies for CyTOF.
