# Supplementary material for: Adipocyte Gata3 Is a Diet-Inducible Driver of Pathological Adipose Remodeling and Systemic Metabolic Disease
Source: Cells. 2026 Sep 10;15(18):1644. doi: 10.3390/cells15181644 (PMC13605690; doi:10.3390/cells15181644)
Supplement: Supplementary file 1 [file cells-15-01644-s001.zip › Method Supplement-9-9-26.pdf]

Methods

Study design

This study was designed to define the cell-autonomous role of *Gata3* in mature adipocytes during diet-induced obesity and to determine whether *Gata3* functions as a necessary, sufficient, or associative regulator of pathological adipose tissue remodeling. To distinguish loss- from gain-of-function effects while minimizing potential biases associated with single-direction genetic models, we employed two complementary adipocyte-specific mouse models: an adipocyte-specific *Gata3* knockout (aGata3KO) model and an adipocyte-specific humanized *GATA3* knock-in (aGATA3KI) model. Both models were evaluated under standard chow and high-fat diet (HFD) conditions to characterize the progression from metabolic homeostasis to established obesity-induced dysfunction.

Unless otherwise indicated, all experiments were performed in male mice. Mice were maintained on either standard chow or HFD for up to 16 weeks before metabolic, molecular, histological, and multi-omics analyses. Individual animals constituted the experimental unit for all in vivo experiments, including proteomic, metabolomic, lipidomic, and multiplex cytokine analyses. For CyTOF experiments, stromal vascular fractions from three biological replicates per experimental group were pooled to obtain sufficient cell numbers for high-dimensional immune profiling. Sample sizes were determined a priori using published variance estimates for metabolic phenotyping studies to achieve 80% statistical power at a two-sided significance level of $\alpha = 0.05$. Investigators were aware of genotype during colony management and breeding but remained blinded during tissue processing, histological evaluation, image quantification, and primary data analysis. No animals or datasets were excluded from analysis. All animal procedures were approved by the Institutional Animal Care and Use Committee (IACUC) and were performed in accordance with the National Institutes of Health Guide for the Care and Use of Laboratory Animals.

Generation of adipocyte-specific *Gata3* knockout mice

Adipocyte-specific *Gata3* knockout (aGata3KO) mice were generated by crossing homozygous *Gata3*-floxed mice, in which loxP sites flank exons 4 and 5 of the murine *Gata3* locus, with hemizygous Adipoq-Cre transgenic mice (Jackson Laboratory, stock no. 010803, Bar Harbor, USA). The floxed *Gata3* strain was obtained through the NIAID/Taconic repository. Adipoq-Cre drives recombination selectively in terminally differentiated adipocytes while sparing stromal

vascular cells, adipocyte progenitors, endothelial cells, and resident immune populations, thereby enabling mature adipocyte-specific deletion of *Gata3*.

Experimental mice carrying Adipoq-Cre and homozygous floxed *Gata3* alleles (aGata3KO) were compared with littermate floxed controls, Adipoq-Cre controls, and heterozygous animals where appropriate. Recombination efficiency and tissue specificity were verified by RT-PCR of fractionated epididymal white adipose tissue. Efficient deletion of *Gata3* was observed in mature adipocytes, whereas expression within the stromal vascular fraction remained unchanged, confirming adipocyte-specific recombination (Fig. 3E).

A constitutive Adipoq-Cre strategy was selected to avoid the metabolic and immunological effects associated with tamoxifen administration, which has been reported to alter adipose tissue inflammation, glucose homeostasis, and systemic metabolism independently of Cre-mediated recombination [1-5].

Generation of adipocyte-specific humanized GATA3 knock-in mice

To determine whether restoration of Gata3 signaling rescues the aGata3KO phenotype and whether human GATA3 can functionally substitute for murine Gata3 in mature adipocytes, we generated an adipocyte-specific humanized knock-in model. ROSA26-STOP–h*GATA3*–IRES–GFP mice (gift of Dr. Maxime Bouchard, McGill University [6]) were crossed with aGata3KO mice (providing Cre driver) to simultaneously delete murine *Gata3* and activate human GATA3 expression exclusively in mature adipocytes.

Dietary challenge and metabolic phenotyping

Male mice were placed on either standard chow (PicoLab, Liverpool, United Kingdom Rodent Diet 20; 5% kcal from fat) or a high-fat diet (Inotiv, West Lafayette, USA, TD88137; 45% kcal from fat containing >60% of total fat-derived calories, 0.2% cholesterol, and 34% sucrose by) beginning at 6–8 weeks of age. High-fat feeding was maintained for 16–20 weeks depending on the experimental endpoint. Animals were housed under controlled environmental conditions (22 ± 1°C; 12-hour light/dark cycle) with unrestricted access to food and water. Body weight was recorded weekly throughout the dietary intervention.

Food intake was quantified during the second week of HFD feeding. Mice were individually housed in cages containing minimal bedding to facilitate recovery of spilled food. Pre-weighed frozen diet pellets were placed on wire mesh inserts, and remaining food together with recovered spillage was weighed every 24 hours for 10 consecutive days starting at 2 weeks post HFD.

Daily food consumption was calculated by subtracting recovered material from the initial food weight.

Glucose and insulin tolerance tests

Glucose tolerance tests (GTTs) were performed following a 16-hour overnight fast. Mice received an intraperitoneal injection of D-glucose (2 g kg^{-1} body weight; Sigma-Aldrich, St. Louis, USA), and blood glucose concentrations were measured from tail vein blood at 0, 15, 30, 60, 120, and 180 minutes using an AimStrip Plus glucometer (Germaine Laboratories, San Antonio, USA).

Insulin tolerance tests (ITTs) were performed after a 6-hour fast by intraperitoneal administration of regular human insulin (0.75 U kg^{-1} body weight; Humulin R, Eli Lilly, Indianapolis, USA). Blood glucose concentrations were measured at 0, 15, 30, 60, and 120 minutes following injection. GTTs and ITTs were conducted in chow-fed mice and repeated following 16 weeks of HFD, with a minimum interval of six days between tests. Glucose excursion was quantified by calculating the area under the curve (AUC) using the trapezoidal method in GraphPad Prism (v11).

Body composition analysis

At study completion, mice were euthanized under isoflurane anesthesia, and individual tissues were collected for quantitative assessment. Epididymal, inguinal, retroperitoneal, and mesenteric white adipose tissue depots together with interscapular brown adipose tissue, liver, heart, and kidneys were carefully dissected, blotted to remove excess fluid, and weighed to the nearest 0.1 mg. Depot-specific tissue weights were analyzed individually to characterize regional changes in adiposity.

Serum biochemistry

Blood was collected at euthanasia by cardiac puncture, and serum was isolated by centrifugation. Total cholesterol concentrations were measured using an enzymatic colorimetric Cholesterol E assay (Fisher Scientific, Pittsburgh, USA). Serum leptin and adiponectin concentrations were determined using commercially available ELISA kits (R&D Systems, Minneapolis, USA and Sigma-Aldrich, Burlington, USA respectively) according to the manufacturers' instructions.

Histology and adipocyte morphometry

Adipose tissue depots were fixed in 4% paraformaldehyde for 24 hours, processed through graded ethanol, embedded in paraffin, and sectioned at a thickness of 5 μm. Sections were stained with hematoxylin and eosin using standard histological procedures.

Adipocyte morphology was quantified from digitized images using Fiji (ImageJ). At least 100 adipocytes per animal were analyzed from multiple non-overlapping microscopic fields. Adipocyte cross-sectional area and diameter were measured, and mean adipocyte size together with adipocyte density within a defined tissue area were calculated for each specimen.

Isolation of stromal vascular fraction cells and in vitro adipocyte differentiation

Stromal vascular fraction (SVF) cells were isolated from epididymal (visceral) or inguinal (subcutaneous) adipose tissue. Excised fat pads were finely minced and digested in HBSS containing collagenase D (2.5 mg ml^{-1}; Sigma-Aldrich, Burlington, USA), dispase (2.5 U ml^{-1}; STEMCELL Technologies, Vancouver, Canada), and 5 mM $CaCl_2$ for 30–40 minutes at 37 °C with gentle agitation. Enzymatic digestion was terminated by addition of DMEM supplemented with 10% fetal bovine serum (FBS) and 1% penicillin-streptomycin. Cell suspensions were filtered through 40-μm strainers and centrifuged to pellet SVF cells. Following erythrocyte lysis using ACK buffer (Thermo Fisher Scientific, Waltham, USA), cells were washed and resuspended in DMEM containing 10% FBS and 1% penicillin-streptomycin for downstream applications.

For adipogenic differentiation, SVF cells were seeded at 1×10^6 cells per well in six-well culture plates. Two days after reaching confluence, differentiation was induced using DMEM containing 10% FBS supplemented with insulin (10 μg ml^{-1}), dexamethasone (1 μM), and 3-isobutyl-1-methylxanthine (IBMX; 0.5 mM). After 48 hours, cultures were maintained in insulin-containing medium alone for an additional six days with medium replacement every two days.

Oil Red O staining

Differentiated adipocytes were washed twice with phosphate-buffered saline (PBS) and fixed in 4% paraformaldehyde for 30 minutes at room temperature. Cells were rinsed with distilled water, equilibrated in 60% isopropanol for 5 minutes, and stained with freshly prepared and filtered Oil Red O working solution for 30 minutes at room temperature. Excess stains were removed by repeated washing with distilled water until the wash solution was clear. Representative bright-field images were acquired following staining. For quantitative analysis, cultures were air-dried completely before dye extraction with 100% isopropanol for 15 minutes. Extracted dye was

transferred to 96-well plates, and absorbance was measured at 492 nm using pure isopropanol as the blank.

RNA extraction and quantitative real-time PCR

Total RNA was isolated from adipose tissue and liver using TRIzol reagent (Thermo Fisher Scientific, Waltham, USA) followed by purification with either the RNeasy Mini Kit or the RNeasy Lipid Tissue Mini Kit (Qiagen, Germantown, USA), depending on tissue type. RNA concentration and purity were determined spectrophotometrically using a NanoDrop instrument. Complementary DNA was synthesized from 1 μg of total RNA using iScript Reverse Transcription Supermix (Bio-Rad, Hercules, USA) according to the manufacturer's protocol. Quantitative PCR was performed in technical triplicate using SsoFast EvaGreen Supermix (Bio-Rad, Hercules, USA) on a Bio-Rad real-time PCR system. Gene expression was normalized to Gapdh, and relative transcript abundance was calculated using the $2^{-\Delta\Delta Ct}$ method. Primer sequences are provided in Table 1. All qPCR data are shown as means ± SD.

Flow cytometry: Epididymal adipose tissue were harvested from the two mice genotypes (Adipoq-Cre and aGata3KO) on high fat diet for 16 weeks. The tissues were digested and signgle cell suspension were used for flow cytometry analysis. Cell viability was determined by incubating cells with LIVE/DEAD® Fixable Blue Dead Cell Stain single-color dyes (Thermo Fisher Scientific, Waltham,USA) for 30 minutes at room temperature. After one rinse with washing buffer, cells were incubated with CD16/32 antibody (Biolegend, San Diego, USA) at 4°C for 15 minutes to block non-specific binding of immunoglobulin to the Fc receptors. Cells were then labeled with the following rat anti-mouse antibodies from Biolegend: PE-labeled anti-CD45, PE-Cy7 labeled anti-CD11b, Brilliant Violet 421-labeled anti-F4/80. Cells were washed twice and resuspended in staining buffer, and immediately recorded with Becton Dickinson LSR Fortessa (BD biosciences, San Jose, USA) at the Flow Cytometry Core of Cedars-Sinai Medical Center. Data were analyzed with Summit V4.3 software. Only singlet cells were analyzed. All doublets were excluded from the analysis by monitoring the SSC/FSC pulse width channel.

Adipose tissue fractionation

To verify adipocyte-specific recombination, adipose tissue was fractionated into mature adipocyte and stromal vascular compartments. Minced adipose tissue was digested with collagenase D (2.5 mg ml^{-1}), dispase (2.5 U ml^{-1}), and 5 mM $CaCl_2$ in HBSS for 30 minutes at

37 °C with gentle agitation. Enzymatic activity was terminated by addition of DMEM containing 10% FBS.

The digested suspension was first filtered through a 250-μm mesh and allowed standing for 5 minutes to permit flotation of mature adipocytes. The floating adipocyte fraction was collected and washed twice in culture medium. The infranatant was subsequently filtered through a 40-μm cell strainer, centrifuged, and treated with ACK lysis buffer to remove erythrocytes. Following washing, stromal vascular cells were resuspended in PBS for downstream analyses. Both fractions were either processed immediately or snap-frozen in liquid nitrogen for subsequent molecular analyses. Cell viability was assessed by trypan blue exclusion, and preparations with viability greater than 85% were used for downstream experiments.

Adipose tissue proteomics

Global proteomic profiling was performed at the UCLA Proteome Research Center. Epididymal white adipose tissue (eWAT) was collected from Adipoq-Cre control and aGata3KO mice after 16 weeks of HFD (n = 5 per group). Approximately 30 mg of tissue was homogenized in lysis buffer containing 4% SDS, 50 mM Tris-HCl (pH 8.0), protease inhibitor cocktail, and phosphatase inhibitor cocktail (Sigma-Aldrich, Burlington, USA) using probe sonication (three 10-second pulses at 30% amplitude on ice). Lysates were clarified by centrifugation (16,000 × g, 20 minutes, 4 °C), and protein concentrations were determined prior to normalization.

Proteins were reduced with tris(2-carboxyethyl)phosphine (5 mM), alkylated with iodoacetamide (10 mM), purified using the protein aggregation capture (PAC) workflow [7], and digested overnight at 37 °C with Lys-C and trypsin. Peptides were desalted, vacuum-dried, and reconstituted in 5% formic acid before LC-MS/MS analysis.

Peptide separation was performed on a Vanquish Neo UHPLC system coupled to an Orbitrap Astral mass spectrometer (Thermo Fisher Scientific, Waltham, USA) using a trap-and-elute configuration with an IonOpticks C18 reverse-phase column (8 cm × 150 μm, 1.7 μm particle size) maintained at 52 °C. Mobile phase A consisted of 0.1% formic acid in water and mobile phase B consisted of 0.1% formic acid in acetonitrile. Peptides were separated using a 15-minute gradient.

Data-independent acquisition (DIA) was performed in positive ion mode. Full MS1 scans were acquired at 240,000 resolution over an m/z range of 380–980. Sequential 4 m/z isolation

windows were used for DIA acquisition, and MS2 spectra were acquired at 80,000 resolution using higher-energy collisional dissociation.

Raw data were processed using DIA-NN with an in silico spectral library generated from the *Mus musculus* reference proteome (UniProt UP000000589) [8]. Protein abundance tables were analyzed in FragPipe Analyst using the Bioconductor limma package [9]. Differentially abundant proteins were identified using an adjusted P value ≤ 0.05 and an absolute $\log_2$ fold change ≥ 1.

Adipose tissue metabolomics

Untargeted metabolomic profiling was performed by the Cedars-Sinai Metabolomics Core Facility using snap-frozen eWAT collected from control and aGata3KO mice after 16 weeks of HFD (n = 3 per group). Approximately 25 mg of tissue was homogenized in ice-cold extraction solvent (40% acetonitrile, 40% methanol, and 20% water), vortexed for 15 minutes at 4 °C, centrifuged (10,000 × g, 10 minutes, 4 °C), and the supernatant was dried under vacuum. Samples were reconstituted in methanol followed by water immediately before analysis. Metabolites were analyzed using an Agilent 1290 UHPLC coupled to an Agilent 6470A triple quadrupole mass spectrometer operating in negative electrospray ionization mode. Chromatographic separation was performed on a ZORBAX RRHD Extend-C18 column with a matching guard column using tributylamine-based ion-pairing chromatography. Six microliters of each extract were injected, and metabolites were separated using a multistep solvent gradient optimized for polar metabolite detection.

Targeted dynamic multiple reaction monitoring (dMRM) was performed using the Agilent MassHunter Metabolomics Database and Method, enabling detection of up to 219 polar metabolites. Instrument control and data acquisition were performed using MassHunter Workstation software. Peak integration was manually reviewed to ensure consistent chromatographic alignment and quantification. Processed metabolite intensities were analyzed using MetaboAnalyst 5.0 following median normalization. Differential metabolite abundance between experimental groups was determined using the statistical workflow implemented within MetaboAnalyst.

Single-cell mass cytometry (CyTOF)

High-dimensional immune profiling was performed by single-cell mass cytometry (CyTOF) in collaboration with the UCLA Jonsson Comprehensive Cancer Center and the UCLA Center for AIDS Research (CFAR) Flow Cytometry Core Facility, essentially as described [10]. Gonadal

white adipose tissue (eWAT) and inguinal white adipose tissue (iWAT) were harvested from three male mice per sample, pooled, finely minced, and enzymatically digested in collagenase D (1.0 mg/mL; Roche, Basel, Switzerland, Cat# 1108874103) and Dispase II (5 mg/mL; STEMCELL Technologies, Vancouver, Canada, Cat# 07913) at 37°C with continuous shaking for 40–50 min. Enzymatic digestion was terminated by adding an equal volume of complete DMEM containing 10% fetal bovine serum (FBS). Cell suspensions were centrifuged at 700 × g for 10 min, resuspended in complete medium, subjected to red blood cell lysis, and filtered through a 100-μm cell stra iner to isolate the stromal vascular fraction (SVF). SVF cells were washed and resuspended in Maxpar Cell Staining Buffer (CSB; Standard BioTools, Boston, USA). Approximately 3 × 10^6 cells in 50 μL CSB were transferred to 5-mL polypropylene tubes for antibody staining. Cell viability was assessed by trypan blue exclusion, and only samples with >80% viability were processed further.

Cell-ID Cisplatin viability staining

Cells were washed once with Maxpar PBS (Standard BioTools, Boston, USA, Cat# 201058) and centrifuged at 300 × g for 5 min. Cell viability was assessed using Cell-ID Cisplatin (Standard BioTools, Boston, USA, Cat# 201064) at a final dilution of 1:5,000 (1 μL of 5 mM cisplatin in 5 mL PBS). Cells were incubated for 5 min at room temperature, after which staining was quenched with at least five volumes of CSB. Cells were washed three times with CSB by centrifugation at 300 × g for 5 min and the washing was twice.

Fc blocking and cell surface staining

Fc receptors were blocked by adding anti-mouse CD16/32 (Biolegend, San Diego, USA, Cat# 101301,) to each tube and incubated for 10 minutes at room temperature. Cocktail of total 33 metal isotope labeled antibodies in 50 μL CSB was added to each tube without washing of the Fc Receptor Blocking Solution. Antibody clones, manufacturers and dilutions for the antibodies are included in Table 2. Some antibodies were labeled in our lab by using Maxpar X8 Antibody Labeling Kit and Maxpar MCP9 Antibody Labeling Kit (Standard BioTools, Boston, USA), which are shown in the attached table as self-labeled. The cells were gently mixed by pipetting and incubated at room temperature for 15 min, samples vortexed gently and incubated for an additional 15 min at room temperature. Cells were washed three times by adding 2 mL CSB to each tube, centrifuging at 300 x g for 5 minutes. After final wash/aspiration, cells were resuspended in residual volume by gently vortex.

Cell fixation and DNA intercalation

Cells were fixed by adding 1 mL freshly prepared 1.6% formaldehyde (prepared from 16% methanol-free formaldehyde; Thermo Fisher Scientific, Waltham, USA, Cat# 28906) in PBS and incubated for 10 min at room temperature. Following fixation, cells were centrifuged at 800 × g for 5 min and resuspended in Maxpar Fix and Perm Buffer containing 125 nM Cell-ID Intercalator-Ir (Standard BioTools, Boston, USA, Part No. 201192A). Samples were incubated overnight at 4°C to label cellular DNA. On the following day, cells were centrifuged at 800 × g for 5 min, washed once with CSB and once with Milli-Q water, and resuspended in 100 μL Milli-Q water. Samples were maintained on ice or at 4°C until acquisition. Final sample preparation for acquisition was completed by the UCLA Flow Cytometry Core Facility according to Standard BioTools recommendations. Data were acquired on a Helios mass cytometer (Standard BioTools) and exported as FCS files for downstream analysis.

Serum lipidomics

Serum lipidomic profiling was performed at the UCLA Lipidomics Core Facility. Serum samples (25 μL) from two control and two aGata3KO groups of mice (n = 3 mice in each group/genotype) were subjected to lipid extraction using a modified Bligh and Dyer protocol. Before extraction, each sample was spiked with a mixture of 74 isotopically labeled internal standards consisting of the Ultimate SPLASH ONE mix supplemented with the SPLASH Booster (Avanti Polar Lipids, Alabama,USA) to enable quantitative normalization across lipid classes.

Following two sequential organic solvent extractions, pooled organic phases were dried under vacuum using a Thermo SpeedVac concentrator and resuspended in methanol:dichloromethane (1:1, v/v) containing 10 mM ammonium acetate. Lipid species were quantified using a SCIEX Triple Quad 6500+ mass spectrometer equipped with differential mobility spectrometry (DMS). Targeted analyses were performed using an expanded multiple reaction monitoring (MRM) method covering approximately 1,450 lipid species across 17 lipid subclasses. Instrument calibration and DMS optimization were performed using the EquiSPLASH LIPIDOMIX standard (Avanti Polar Lipids, Alabama, USA). Quantitative data processing was conducted using an in-house analysis pipeline comparable to the Lipidyzer Workflow Manager. Instrument settings, MRM transitions, analytical workflows, and method validation have been described previously [11]. Lipid abundances were normalized to serum volume before statistical analysis.

Multiplex serum cytokine and chemokine profiling

Circulating cytokines and chemokines were quantified using the MILLIPLEX® MAP Mouse Cytokine/Chemokine Magnetic Bead Panel (32-plex; MilliporeSigma, Burlington, USA) according to the manufacturer's instructions. Fluorescence data were acquired on a Luminex® 200 platform, and analyte concentrations were calculated from standard curves using Milliplex Analyst software (version 5.1). All assays were performed by the UCLA Immune Assessment Core Facility.

The multiplex panel quantified the following analytes: CCL11 (Eotaxin), G-CSF, GM-CSF, IFN-γ, IL-1α, IL-1β, IL-2, IL-3, IL-4, IL-5, IL-6, IL-7, IL-9, IL-10, IL-12p40, IL-12p70, IL-13, IL-15, IL-17A, CXCL10 (IP-10), CXCL1 (KC), LIF, CXCL5 (LIX), CCL2 (MCP-1), M-CSF, CXCL9 (MIG), CCL3 (MIP-1α), CCL4 (MIP-1β), CXCL2 (MIP-2), CCL5 (RANTES), TNF-α, and VEGF. Analytes detected in only a small proportion of samples or failing predefined quality-control criteria were excluded from downstream statistical analyses.

Statistical analysis

Statistical analyses were performed using GraphPad Prism (version 11.0) unless otherwise specified within individual methodology sections. Data are presented as mean ± s.e.m. unless indicated otherwise.

Data distributions were evaluated using the Shapiro–Wilk normality test. Comparisons between two groups were performed using two-tailed unpaired Student's *t*-tests for normally distributed data or Mann–Whitney *U* tests for nonparametric data. Longitudinal measurements and experiments involving multiple groups were analyzed using two-way repeated-measures analysis of variance (ANOVA) followed by Tukey's multiple-comparison test.

For high-dimensional datasets, statistical analyses and multiple-testing correction were performed using the analysis pipelines described in the corresponding methodology sections. Differential protein abundance was determined using the limma package with Benjamini–Hochberg false discovery rate correction, whereas CyTOF population frequencies were analyzed using two-proportion *z*-tests with false discovery rate adjustment. Statistical thresholds for metabolomic and lipidomic datasets are described within their respective analytical workflows.

Unless otherwise specified, statistical significance was defined as $P < 0.05$. Significance levels are denoted as follows: $P < 0.05$ (*), $P < 0.01$ (**), $P < 0.001$ (***), *and* $P < 0.0001$ (****). Sample sizes were determined as described under Study design. Animals were assigned to

experimental groups according to genotype. Investigators were blinded during tissue processing, image analysis, histological quantification, and primary data analysis but not during colony management or animal husbandry. No animals or datasets were excluded from analysis. Unless otherwise indicated, all experiments were performed using at least three independent biological replicates.

References

[1] Liu Z, Cheng Y, Luan Y, Zhong W, Lai H, Wang H, et al. Short-term tamoxifen treatment has long-term effects on metabolism in high-fat diet-fed mice with involvement of Nmnat2 in POMC neurons. FEBS Letters. 2018;592:3305-16.

[2] Stout MB, Scalzo RL, Wellberg EA. Persistent Metabolic Effects of Tamoxifen: Considerations for an Experimental Tool and Clinical Breast Cancer Treatment. Endocrinology. 2021;162.

[3] Estrada-Meza J, Videlo J, Bron C, Saint-Béat C, Silva M, Duboeuf F, et al. Tamoxifen Treatment in the Neonatal Period Affects Glucose Homeostasis in Adult Mice in a Sex-Dependent Manner. Endocrinology. 2021;162:bqab098.

[4] Liu L, Zou P, Zheng L, Linarelli LE, Amarell S, Passaro A, et al. Tamoxifen reduces fat mass by boosting reactive oxygen species. Cell Death & Disease. 2015;6:e1586-e.

[5] Zhao L, Wang B, Gomez NA, de Avila JM, Zhu M-J, Du M. Even a low dose of tamoxifen profoundly induces adipose tissue browning in female mice. International Journal of Obesity. 2020;44:226-34.

[6] Nguyen AHT, Tremblay M, Haigh K, Koumakpayi IH, Paquet M, Pandolfi PP, et al. Gata3 antagonizes cancer progression in Pten-deficient prostates. Human Molecular Genetics. 2013;22:2400-10.

[7] Batth TS, Tollenaere MX, Rüther P, Gonzalez-Franquesa A, Prabhakar BS, Bekker-Jensen S, et al. Protein Aggregation Capture on Microparticles Enables Multipurpose Proteomics Sample Preparation. Molecular & cellular proteomics : MCP. 2019;18:1027-35.

[8] Demichev V, Messner CB, Vernardis SI, Lilley KS, Ralser M. DIA-NN: neural networks and interference correction enable deep proteome coverage in high throughput. Nat Methods. 2020;17:41-4.

[9] Hsiao Y, Zhang H, Li GX, Deng Y, Yu F, Valipour Kahrood H, et al. Analysis and Visualization of Quantitative Proteomics Data Using FragPipe-Analyst. J Proteome Res. 2024;23:4303-15.

[10] Shin SM, Hernandez A, Coyne E, Munjal K, Gross NE, Charmsaz S, et al. CyTOF protocol for immune monitoring of solid tumors from mouse models. STAR Protoc. 2023;4:101949.

[11] Su B, Bettcher LF, Hsieh WY, Hornburg D, Pearson MJ, Blomberg N, et al. A DMS Shotgun Lipidomics Workflow Application to Facilitate High-Throughput, Comprehensive Lipidomics. J Am Soc Mass Spectrom. 2021;32:2655-63.

Table S1
List of qPCR primers

**Gene**	Forward 5'−3'	Reverse 5'−3'
Gata3	GAAATACTGTACATTTGAGGAA	TTGGACATCAGACTTAGTGGT
Pparγ	GCTGTCATTATTCTCAGTGGAGAC	CAGGTTGTCTTGGATGTCCTC
C/ebpα	GCAAAGCCAAGAAGTCGGTGGA	CCTTCTGTTGCGTCTCCACGTT
Scd-1	CCTGCGGATCTTCCTTATCATT	GTGGGCGCGGTGATCTC
Srebp-1c	CGACTACATCCGCTTCTTGCAG	CCTCCATAGACACATCTGTGCC
Perilipin 2	GACAGGATGGAGGAAAGACTGC	GGTAGTCGTCACCACATCCTTC
Perilipin 5	GCCACAGTGAATGATCTTGCCTG	GTCCACCATGCCTGTGACACTT
Lptin	GCAGTGCCTATCCAGAAAGTCC	GGAATGAAGTCCAAGCCAGTGAC
Adiponectin	AGATGGCACTCCTGGAGAGAAG	ACATAAGCGGCTTCTCCAGGCT
IL-6	ACAAAGCCAGAGTCCTTCAGAG	AGCCACTCCTTCTGTGACTCC
IGF-1	TGACATGCCCAAGACTCAGA	AGGTTGCTCAAGCAGCAAAG
IL1-β	TGGACCTTCCAGGATGAGGACA	GTTCATCTCGGAGCCTGTAGTG
TNFR1	GTGTGGCTGTAAGGAGAACCAG	CACACGGTGTTCTGAGTCTCCT
TNFR2	TGACAGGAAGGCTCAGATGTGC	ATGCTTGCCTCACAGTCCGCAC
Lox	TGGCTATGGGAGAATGGAAC	GCTCCGTCTTGAAGGTATGC
TGF-β	GTGACAGCAAAGATAACAAACTCC	GTGAGCTGTGCAGGTGCTG
Col1a1	CCTCAGGGTATTGCTGGACAAC	CAGAAGGACCTTGTTTGCCAGG
Col3a1	GACCAAAAGGTGATGCTGGACAG	CAAGACCTCGTGCTCCAGTTAG
Col6a1	GACACCTCTCAGTGTGCTCTGT	GCGATAAGCCTTGGCAGGAAATG
Stat3	AGGAGTCTAACAACGGCAGCCT	GTGGTACACCTCAGTCTCGAAG
Pcsk9	AACCTGGAGCGAATTATCCCA	TTGAAGTCGGTGATGGTGACC
Apoa1	TATGTGGATGCGGTCAAAGA	CAGAAGTCCCGAGTCAATGG

Table S2.

List of Antibodies for CyTOF

No.	Target	Label	Dilution	Clone	Catalog#	Vendor
1	CD45	89Y	1:100	30-F11	3089005B	Fluidigm
2	Ly-6C	116Cd	1:100	HK1.4	128039 (Self-labeled)	Biolegend
3	CD44	165Ho	1:100	IM7	103051 (Self-labeled)	Biolegend
4	CD11c	209Bi	1:100	N418	3209005B	Fluidigm
5	Siglec-F	147Sm	1:200	S17007L	155502 (Self-labeled)	Biolegend
6	I-A/I-E	174Yb	1:400	M5/114.15.2	3174003B	Fluidigm
7	CD140b	151Eu	1:50	APB5	3151017B	Fluidigm
9	CD11b	172Yb	1:200	M1/70	3172012B	Fluidigm
10	CD19	166Er	1:100	6D5	3166015B	Fluidigm
11	CD24	150Nd	1:100	M1/69	3150009B	Fluidigm
12	CD31	160Gd	1:50	390	102425 (Self-labeled)	Biolegend
13	CD26	154Sm	1:100	H194-112	137801 (Self-labeled)	Biolegend
14	CD54 (ICAM-1)	163Dy	1:200	YN1/1.7.4	3163020B	Fluidigm
15	CD117 (ckit)	173Yb	1:50	2B8	3173004B	Fluidigm
16	CD140a	148Nd	1:50	APA5	3148018B	Fluidigm
17	CD64	161Dy	1:50	X54-5/7.1	139301 (Self-labeled)	Biolegend
18	CD142	153Eu	1:50	Polyclonal Goat IgG	AF3178 (Self-labeled)	R&D
19	CD25 (IL-2R)	156Gd	1:50	3C7	101913 (Self-labeled)	Biolegend
20	CD8b	146Nd	1:50	H35-17.2	14-0083-82 (Self-labeled)	ThermoFisher
21	Ly-6A/E (Sca-1)	164Dy	1:500	D7	3164015B	Fluidigm
22	CD4	145Nd	1:50	RM4-5	3145002B	Fluidigm
23	CD9	158Gd	1:200	KMC8	3158009B	Fluidigm
24	Ly-6G	141Pr	1:100	1A8	3141008B	Fluidigm
25	KLRG1	176Yb	1:50	2F1	562190 (Self-labeled)	BD Pharmingen™

26	TCRb	143Nd	1:50	H57-597	3143010B	Fluidigm
27	CD161 (NK1.1)	170Er	1:50	PK136	3170002B	Fluidigm
28	CD29	167Er	1:100	HMβ1-1	102201 (Self-labeled)	Biolegend
29	CD206 (MMR)	169Tm	1:100	C068C2	3169021B	Fluidigm
30	CD34	149Sm	1:50	RAM34	14-0341-82 (Self-labeled)	eBioscience
31	CD3e	152Sm	1:50	145-2C11	3152004B	Fluidigm
32	CD127 (IL-7Ra)	175Lu	1:50	A7R34	3175006B	Fluidigm
33	CD45R (B220)	144Nd	1:100	RA3-6B2	3144011B	Fluidigm
