# Supplementary material for: Adipocyte Gata3 Is a Diet-Inducible Driver of Pathological Adipose Remodeling and Systemic Metabolic Disease
Source: Cells. 2026 Sep 10;15(18):1644. doi: 10.3390/cells15181644 (PMC13605690; doi:10.3390/cells15181644)
Supplement: Supplementary file 1 [file cells-15-01644-s001.zip › Figure Supplement-9-9-26.pdf]

GO:BP		stats
Term name	Term ID	p_{adj}
immune system process	GO:0002376	8.917×10^{-19}
phagocytosis	GO:0006909	1.127×10^{-18}
immune response	GO:0006955	7.073×10^{-15}
regulation of immune system process	GO:0002682	5.393×10^{-14}
inflammatory response	GO:0006954	3.042×10^{-13}
cell activation	GO:0001775	3.571×10^{-13}
endocytosis	GO:0006897	2.101×10^{-12}
defense response	GO:0006952	5.007×10^{-12}
positive regulation of immune system process	GO:0002684	6.234×10^{-12}
leukocyte activation	GO:0045321	1.971×10^{-11}
response to stress	GO:0006950	4.040×10^{-11}
response to stimulus	GO:0050896	5.690×10^{-11}
import into cell	GO:0098657	2.587×10^{-10}
positive regulation of biological process	GO:0048518	2.729×10^{-10}
vesicle-mediated transport	GO:0016192	4.178×10^{-10}
cell migration	GO:0016477	5.613×10^{-10}
immune effector process	GO:0002252	6.350×10^{-10}
response to external stimulus	GO:0009605	1.162×10^{-9}
cellular response to stimulus	GO:0051716	1.345×10^{-9}
positive regulation of response to stimulus	GO:0048584	2.096×10^{-9}
leukocyte migration	GO:0050900	2.235×10^{-9}
cell motility	GO:0048870	3.081×10^{-9}
regulation of phagocytosis	GO:0050764	3.602×10^{-9}
defense response to other organism	GO:0098542	5.166×10^{-9}
regulation of response to stimulus	GO:0048583	9.125×10^{-9}
response to biotic stimulus	GO:0009607	1.044×10^{-8}
lymphocyte activation	GO:0046649	1.143×10^{-8}
cytokine production	GO:0001816	1.170×10^{-8}
regulation of immune response	GO:0050776	1.888×10^{-8}
response to other organism	GO:0051707	2.191×10^{-8}
response to external biotic stimulus	GO:0043207	2.341×10^{-8}

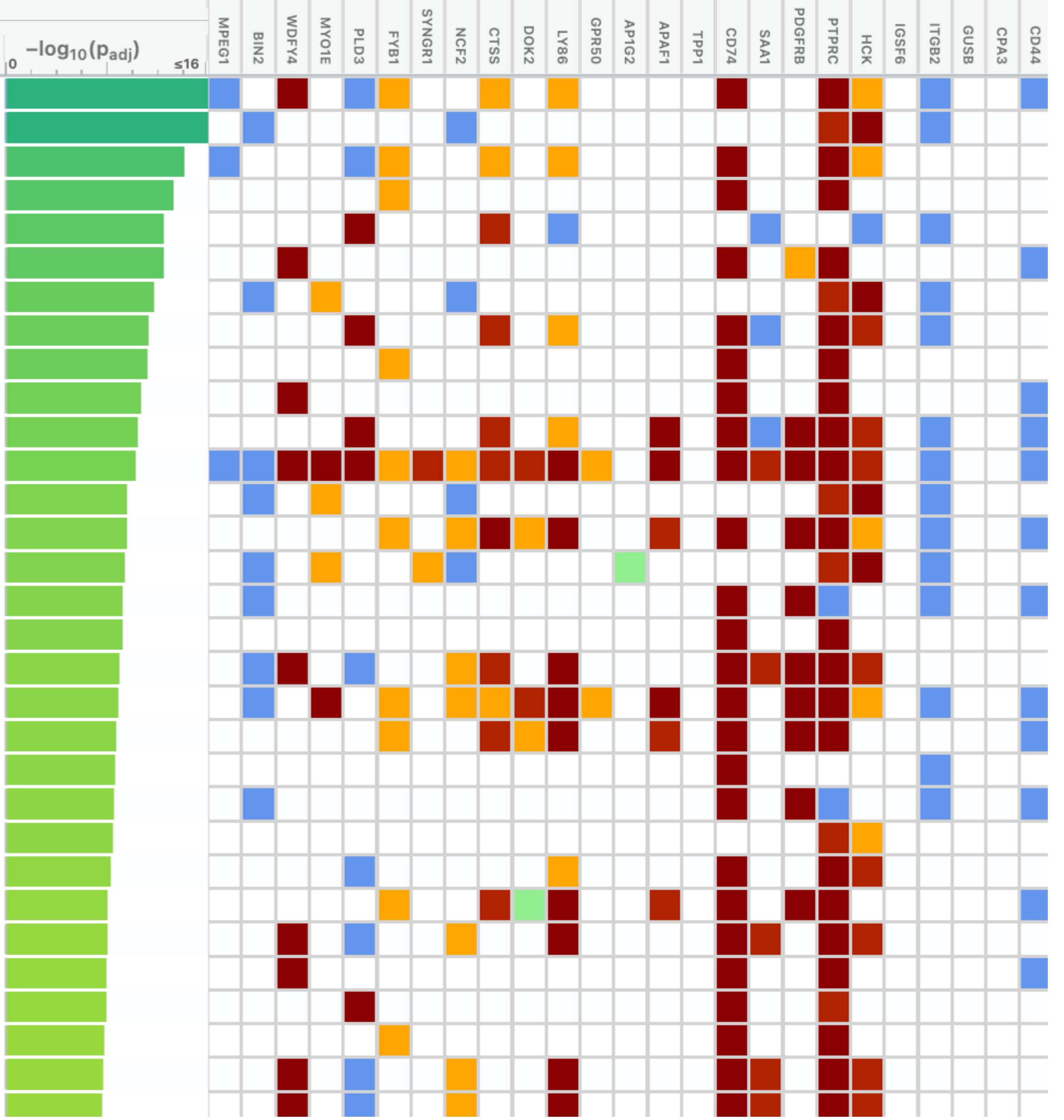

Figure S1 | g:Profiler pathway-enrichment analysis of adipose-tissue proteomic changes in aGata3KO mice. Differential proteomic data from adipose tissue of adipocyte-specific Gata3 knockout (aGata3KO) mice were analyzed using gProfiler, as described in the Methods. A truncated view of the gProfiler output is shown to improve readability. Immune system–related pathways were prominently affected, indicating that adipocyte Gata3 deficiency substantially alters immune-associated processes in adipose tissue.

Percentage of CD11b & F4/80 positive cells in total single cells of SVF (excluded neutrophil and eosino[illegible]

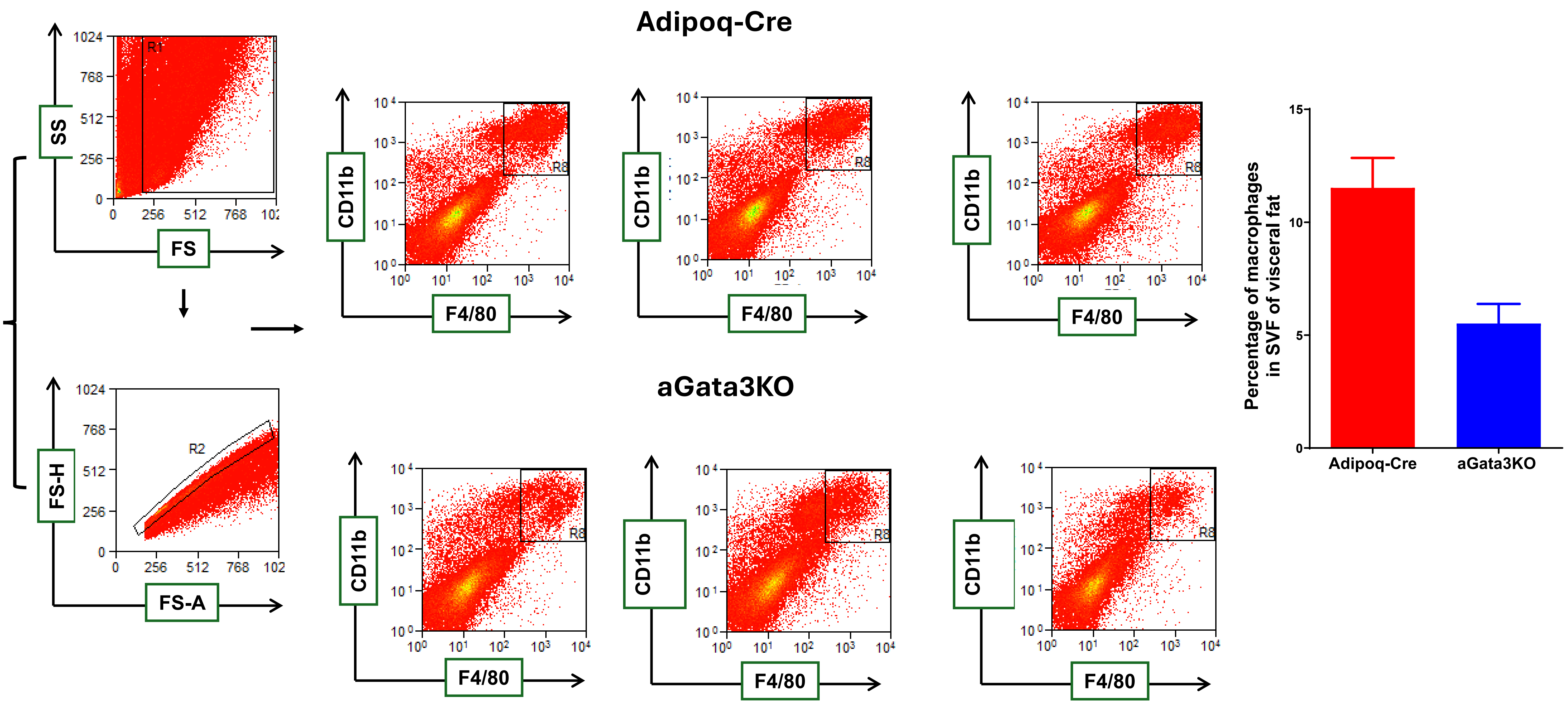

Fig. S2. Adipocyte-specific Gata3 deficiency reduces macrophage content in epididymal adipose tissue. Adipoq-Cre control and adipocyte-specific Gata3 knockout (aGata3KO) mice were fed a high-fat diet (HFD) for 16 weeks. Epididymal adipose tissue was then harvested and processed for flow-cytometric analysis as described in the Methods (n = 3 mice per genotype). Stromal vascular cell suspensions were stained with the indicated antibodies and analyzed using the indicated gating strategy. Each data point represents epididymal adipose tissue from an individual mouse. Statistical comparisons were performed using a two-tailed Student's *t*-test.

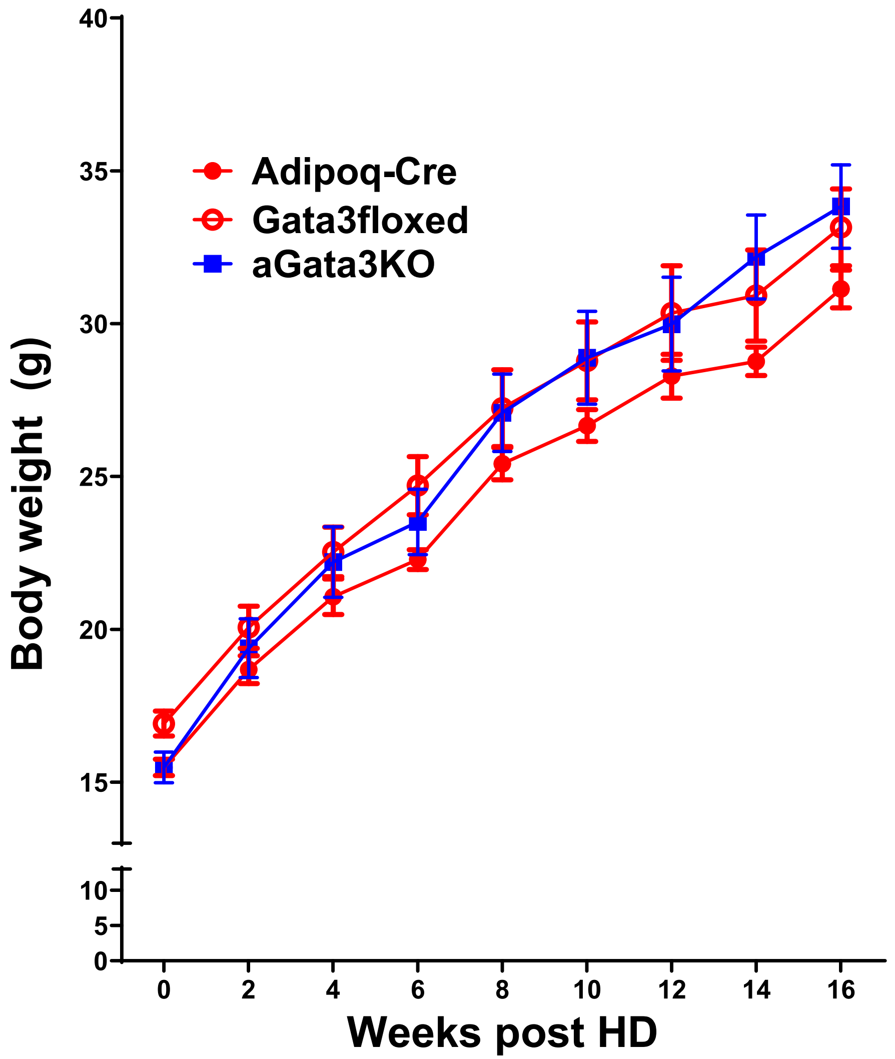

Fig. S3. Gata3 deficiency does not alter weight gain in female mice fed a high-fat diet. Female control mice (Adipoq-Cre and Gata3^floxed^) and adipocyte-specific Gata3 knockout (aGata3KO) mice were maintained on a high-fat diet (HFD) for 16 weeks. Body-weight gain was assessed as described in the Methods (n = 8–10 mice per genotype). Weight gain did not differ significantly among the groups.
